# A protein signature associated with active tuberculosis identified by plasma profiling and network-based analysis

**DOI:** 10.1016/j.isci.2022.105652

**Published:** 2022-11-22

**Authors:** Zaynab Mousavian, Elin Folkesson, Gabrielle Fröberg, Fariba Foroogh, Margarida Correia-Neves, Judith Bruchfeld, Gunilla Källenius, Christopher Sundling

**Affiliations:** 1 Division of Infectious Diseases, Department of Medicine Solna, Karolinska Institutet, Stockholm, Sweden; 2 Center for Molecular Medicine, Karolinska Institutet, Stockholm, Sweden; 3 School of Mathematics, Statistics and Computer Science, College of Science, University of Tehran, Tehran, Iran; 4 Department of Infectious Diseases, Karolinska University Hospital, Stockholm, Sweden; 5 Department of Clinical Microbiology, Karolinska University Laboratory, Karolinska University Hospital, Stockholm, Sweden; 6 Life and Health Sciences Research Institute, School of Medicine, University of Minho, Braga, Portugal; 7 ICVS/3B’s, PT Government Associate Laboratory, Braga, Portugal

**Keywords:** clinical finding, disease, proteomics, infectious disease

## Abstract

Annually, approximately 10 million people are diagnosed with active tuberculosis (TB), and 1.4 million die of the disease. If left untreated, each person with active TB will infect 10–15 new individuals. The lack of non-sputum-based diagnostic tests leads to delayed diagnoses of active pulmonary TB cases, contributing to continued disease transmission. In this exploratory study, we aimed to identify biomarkers associated with active TB. We assessed the plasma levels of 92 proteins associated with inflammation in individuals with active TB (n = 20), latent TB (n = 14), or healthy controls (n = 10). Using co-expression network analysis, we identified one module of proteins with strong association with active TB. We removed proteins from the module that had low abundance or were associated with non-TB diseases in published transcriptomic datasets, resulting in a 12-protein plasma signature that was highly enriched in individuals with pulmonary and extrapulmonary TB and was further associated with disease severity.

## Highlights

• We identified a 12-protein signature highly associated with active tuberculosis (TB)
• The signature was correlated with TB disease severity and inflammation
• The signature was enriched in active TB compared with other diseases
• IFNG, IL-6, and VEGFA of the signature have previously been associated with active TB

## Graphical abstract

**Figure:**
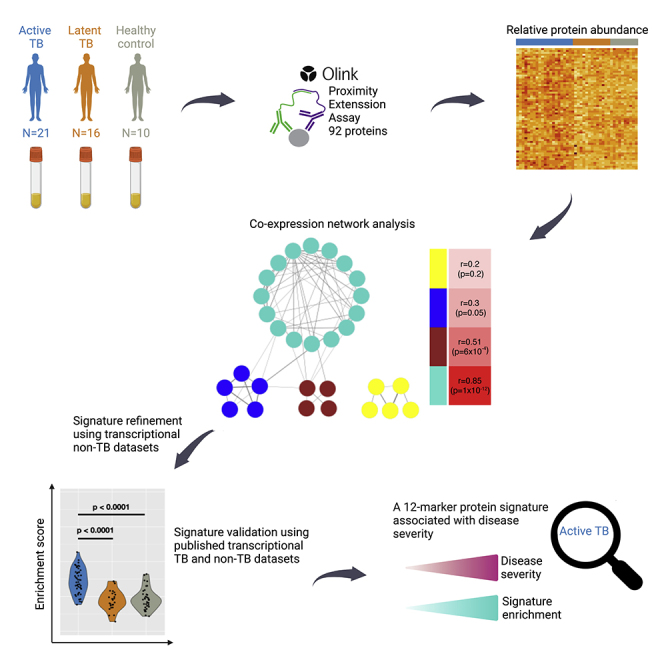

## Introduction

Tuberculosis (TB), caused by *Mycobacterium tuberculosis* (Mtb), is an ongoing pandemic responsible for approximately 10 million clinical cases and 1.4 million deaths annually, making it the single deadliest infectious disease excluding the ongoing SARS-CoV2 pandemic. However, there are still many limitations in diagnostic methods available for active TB.^1^^,^^2^ Around one-fourth to one-fifth of the world’s population is estimated to be latently infected with Mtb,^3^ of which 5%–10% are estimated to eventually develop active disease.

Pulmonary TB is the most common clinical form, and it is diagnosed by detecting Mtb in sputum samples by microscopy, nucleic acid amplification tests, such as PCR, and the reference method mycobacterial culture. Several limitations to these methods exist; as compared with culture positivity, sputum microscopy is positive only in approximately 50% of cases^4^ and PCR in approximately 90% of cases in respiratory samples (GeneXpert RIF/TB and GeneXpert Ultra).^5^^,^^6^ Additional difficulties relate to specific patient groups, such as HIV-infected patients and children, the latter commonly having low bacterial load and cannot produce sputum. Mycobacterial culture can take several weeks to yield positive results and requires specialized safety laboratory conditions. In addition, it is often unavailable in resource-poor settings where TB is more prevalent. In addition, the diagnosis of extrapulmonary TB relies on invasive procedures to obtain samples for microbiological analysis. Thus, diagnosis is frequently based on clinical and radiological findings or algorithms, especially in low-resource settings, with delays in the diagnosis in endemic areas as a result.^7^ The gap between estimated and reported TB cases was more than 4 million in 2020, and of those reported only 59% were microbiologically confirmed.^8^ No specific blood test or biochemical marker have yet been introduced in the routine clinical workup to distinguish TB from other medical conditions. The need for non-sputum-based tests, both for screening and diagnostic purposes, is urgent, and the requirements of those tests have been described in detail in WHO statements for Target Product Profiles.^9^

Over the last decades, various methods that examine the host response to Mtb infection have been evaluated. Attempts at identifying TB-specific transcriptional, protein, and metabolic signatures in patient blood samples were recently reviewed,^10^^,^^11^ with promising results emerging for transcriptional signatures.^12^^,^^13^ The different protein signatures so far identified show limited overlap, and together with varying study designs and methods, this makes a meta-analysis difficult.^14^^,^^15^ The TB biomarker field is however rapidly expanding, and data-driven methods to broadly characterize the plasma proteome are increasingly used.^15^ The Olink proximity extension assay (PEA), using affinity proteomics to measure relative protein concentrations in biological materials by qPCR,^16^ has gained increasing attention and use in characterizing patterns of proteins in health and disease.^17^^,^^18^^,^^19^

In this study, we used the Olink PEA to profile 92 inflammatory proteins in the plasma from a Swedish cohort including individuals with active TB, latent TB, and healthy controls. Through weighted co-expression network analysis, we identified a signature that was highly associated with active TB and disease severity. We refined the signature by removing proteins associated with other bacterial and viral respiratory infections and sarcoidosis. We then assessed the signature in several independent transcriptional datasets and found that it is highly enriched in individuals with active TB.

## Results

### Clinical characteristics of the study participants

The plasma of 21 individuals with active TB, 16 individuals with latent TB, and 10 healthy controls were analyzed using the Olink inflammation proximity extension assay. The inflammation panel was selected, as it includes many cytokines and chemokines associated with infection and identified previously, but also new candidates not evaluated as biomarkers in TB. During data processing, 3 samples (1 active TB and 2 latent TB) were excluded for failing the quality control check included in the OlinkRPackage. The characteristics of the 44 study participants are further described in Table 1. Two donors (1 active TB and 1 latent TB) were identified as outliers by cluster analysis (Figure S1). These outliers were not included in the co-expression analysis used for biomarker discovery.

As a reflection of the low TB incidence in Sweden, most study individuals originated from other countries, mainly situated in Africa, Asia, and Eastern Europe. Sixteen of 20 active TB cases had pulmonary TB including 3 with pleuritis, 7 of which were sputum smear-positive. Four cases had extrapulmonary TB, of which 2 with disseminated disease. Only 1 out of the 20 active TB patients was not confirmed by microbiological culture; this patient had a positive PCR for *Mtb* in a lymph node aspirate and gastric lavage as well as radiological signs of active pulmonary TB and showed a clinical response to anti-TB treatment. The active TB cases were sampled within 1 week of anti-TB treatment initiation except for 1 patient that was sampled on day 9. Of the 14 latent TB individuals, 10 had a known recent TB exposure 1–4 months prior to study inclusion. All but 2 latent TB individuals completed preventive TB treatment and none progressed to active TB during a follow-up period of >2 years. There were few significant comorbidities, with no patient in either group on immunosuppressive treatment. All active TB patients were HIV-negative. The latent TB individuals and healthy controls were not routinely tested for HIV infection. Of the 10 healthy controls, 1 had a QFT-Gold Plus result in the low range of the borderline interval (0.35–0.99 IU/mL) but with no known TB exposure, potentially indicating a false-positive result.^20^^,^^21^ All groups had a similar gender distribution, but the healthy controls were significantly younger than the active and latent TB groups (Table 1).

**Table 1:** Clinical characteristics of the sample cohort

		Active TB (n = 20)	Latent TB (n = 14)	Healthy controls (n = 10)	Statistics
Men n (%)		9 (45)	8 (57)	6 (60)	gp = 0.8
Age, y (mean, range)		39 (20–71)	39 (18–72)	25 (21-37)	hp = 0.02
Origin from TB high endemic country, n		15	6	0	
Time since immigration to Sweden, y (mean, range)		9.5 (0.2–45)	6 (1–15)	N/A	
BCG-vaccination	yes/no/unknown	2/1/17	4/1/9	1/9/0	
IGRA result	pos/neg/unknown	9/1/10	13/0/0	1^f^/9/0	
Previous Mtb infection	Active^a^	3	0		
	Latent	1	0		
Time since Mtb exposure^d^	<2 years (recent)	3	10		
	>2 years (remote)	2	1		
**Biochemistry**^b^^,^^e^ (mean, range)	CRP (mg/L)	25 (1–94)	1 (1–2)		p = 0.00015
	ESR (mm/h)	54 (7–119)	6 (1–21)		p < 0.0001
	WBC (10^9^/L)	6.4 (3.2–12)	6.6 (5.1–9)		p = 0.465
	Hb (g/L)	125 (102–149)	145 (118–158)		p = 0.0018
	Alb^c^ (g/L)	32 (26–38)	41 (38–44)		p = 0.00019

Patient origins; Active TB: Somalia (4) Eritrea (3), Sweden (3), Philippines (2), Peru, Bangladesh, Pakistan, Mongolia, Poland, China, Afghanistan. Latent TB: Sweden (3), Romania (3), Moldavia (2), Mongolia, Ethiopia, Eritrea, Ghana, India.

Co-morbidities: Active TB: postpartum period, hypertension (2), intestinal schistosomiasis, chronic hepatitis B, acute thyroiditis. Latent TB: chronic hepatitis B, hypertension (2), pregnancy.

CRP = C-reactive protein, ESR = Erythrocyte sedimentation rate, WBC = White blood cell count, Hb = Hemoglobin, Alb = Albumin.

a Two patients previously treated for ATB, 1 and 4 years before; one patient not treated.

b ATB n = 19 LTBI n = 9.

c ATB n = 17 LTBI n = 7.

d Exposure self-reported or verified.

e Normal range; CRP<5, ESR <20, WBC 4.4–10.0, Hb > 120 (F),>130 (M), Alb>38 (Wilcox rank-sum test was used for statistical testing of mean difference between groups).

f Healthy controls; one QFT borderline (0.36/0.39), no exposure to TB.

g Fisher’s exact test.

h Kruskal-Wallis test.

Consistent with clinical disease, the patients with active TB had significantly higher C-reactive protein (CRP) and erythrocyte sedimentation rate (ESR) and lower albumin (Alb) and hemoglobin (Hb), compared with latent TB individuals (Table 1).

### Network construction reveals one module associated with active TB

**Figure 1:**
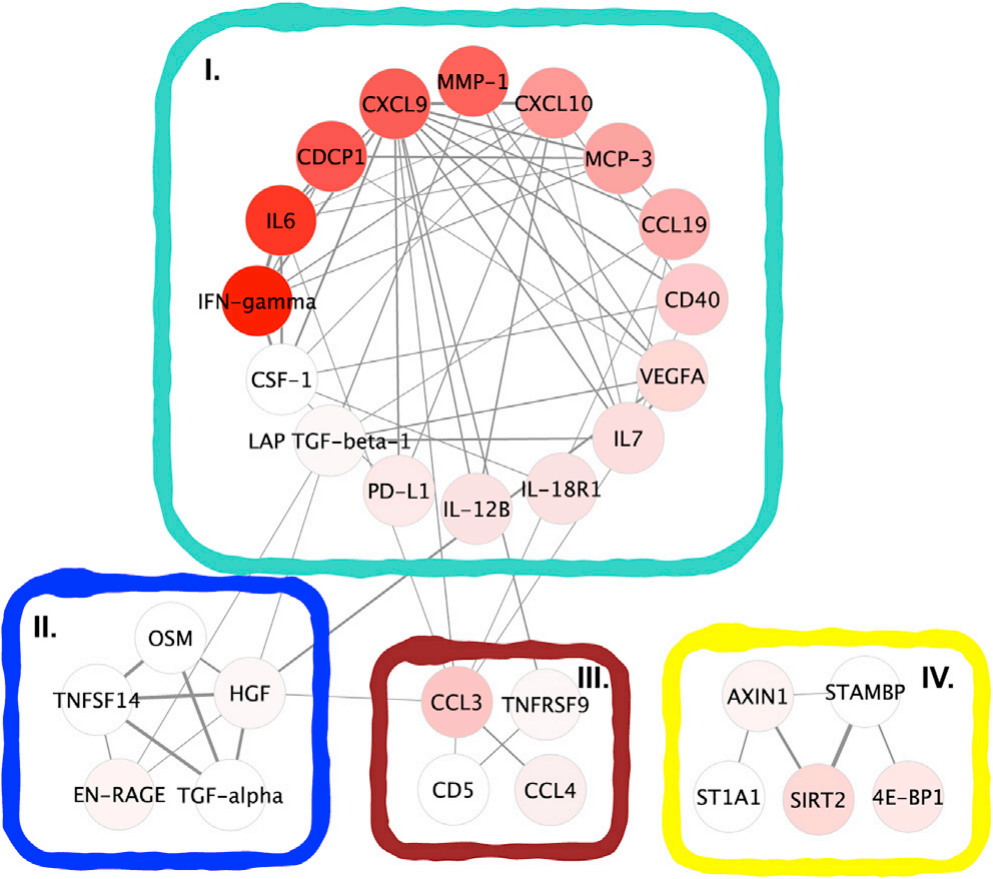
A visual representation of the protein co-expression network analysis and the resulting protein modules, which are indicated by different colors; turquoise (I), blue (II), brown (III), or yellow (IV) Nodes (circles) are colored based on fold-change in protein levels between active TB versus healthy controls, with darker red color indicating a larger fold-change. Links between proteins are depicted by lines, with the thickness proportional to the Pearson correlation coefficient. Only links representing Pearson’s r > 0.6 are shown.

Following data pre-processing, including quality control, batch correction, and clustering, the relative level (NPX) of each of the 92 proteins was assessed. Proteins with more than 30% of samples with NPX values below the limit of detection (n = 28) were excluded from further analysis. Out of those 28 proteins, 25 had no differential expression between the groups (Figure S2). A weighted protein co-expression network was constructed with the remaining 64 proteins to examine correlations between protein expression profiles in all included samples (n = 42 individuals). We considered only the links associated with positive correlations in the network reconstruction and selected power 8 to reach a scale-free topology (Figure S3). After clustering the network into modules, 4 modules indicated by distinct colors were discovered (Figure 1), including 34 proteins. The remaining 30 proteins that were not included in any of the modules were discarded from further analysis. The Pearson correlation between each module eigengene (i.e. the first principal component of each module representing the expression diversity of all proteins in the module) and the active TB phenotype was calculated. We observed that the turquoise module had a better correlation with active TB (r = 0.85, p < 0.0001) compared with the other modules (Blue, r = 0.3, p = 0.05; Brown, r = 0.51, p = 0.0006; Yellow, r = 0.2, p = 0.2). A visual representation of the protein co-expression network and the identified protein modules was then generated using Cytoscape 3.0^22^ (Figure 1).

**Figure 2:**
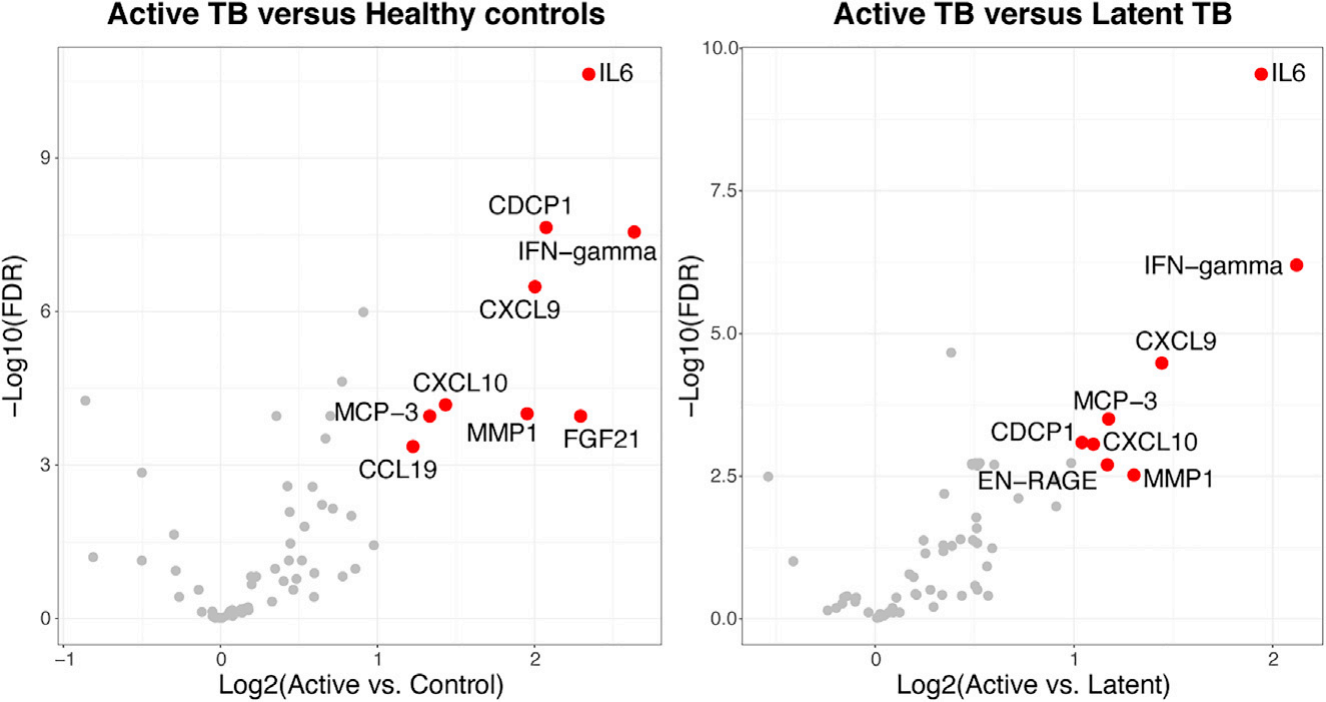
Differential expression analysis of all 64 proteins: active TB versus healthy controls (left) and active TB versus latent TB (right) Red points indicate highly expressed proteins with log2(FC) > 1 and FDR-corrected p-value <0.01 in each comparison.

We then assessed how the markers differed between individuals with active TB, latent TB, and healthy controls (Figure S4). All differentially expressed proteins (log2(FC) ≥ 1 and FDR-corrected p value <0.01) between either active TB and latent TB or active TB and healthy controls except FGF-21 and EN-RANGE were included in the turquoise module (Figure 2). Eight of the 16 proteins in the turquoise module were highly expressed in active TB patients (log2(FC) ≥ 1), whereas the remaining 8 proteins were strongly co-expressed with those proteins. To illustrate how the turquoise module segregated active TB individuals from latent TB and healthy controls, we performed principal component analysis (PCA) on the expression profile of proteins of each module and used the first and the second principal components (PC1 and PC2) to show differences in protein levels (Figure S5). All active TB samples except one were clearly separated from the other samples by the proteins in the turquoise module, suggesting that the included proteins can potentially serve as a signature to identify individuals with active TB.

### Validating the turquoise module in independent transcriptomic TB datasets

There is limited overlap in markers between previous studies investigating protein or gene signatures aiming to discriminate active TB from latent TB, healthy controls, or other diseases (Figure S6). In addition, we did not identify any available pre-processed proteomic dataset including all proteins of the turquoise module that we could use for validation. For this reason, we chose to instead evaluate to what extent the proteins of the different modules could distinguish active TB from latent TB and healthy controls in transcriptomic datasets. We applied all 4 modules to several independent cohorts using multiple gene set enrichment analysis methods. Nine transcriptomic TB datasets were selected based on the criteria of age >15 years, number of individuals per group >10, and no anti-TB treatment (Table S1). The datasets were provided in the curatedTBData R package,^23^ which also included the corresponding genes to all proteins included in the four modules. In total the datasets we selected included >3,000 individuals from 4 continents. Three enrichment methods (ssgsea, gsva, and *Z* score) demonstrated high enrichment of the turquoise module in active TB and low enrichment in latent TB and the control group across most of the TB datasets (Figures S7, S8 and S9). This was also the case for the blue module, although to a less extent compared with the turquoise module. In contrast, neither the brown nor yellow modules were found highly enriched in active TB using the same three methods on the same transcriptomic datasets (Figures S7, S8, and S9).

### Identification of a 12-protein signature for active TB diagnosis

We identified the turquoise module as having the highest correlation with active TB. However, 2 of the 16 proteins in the module, LAP TGF-beta-1 and CSF-1, had low fold-change values (log2(FC) < 0.5) when comparing active TB with both latent TB and healthy controls (Figure 2) and were therefore potentially redundant.

**Figure 3:**
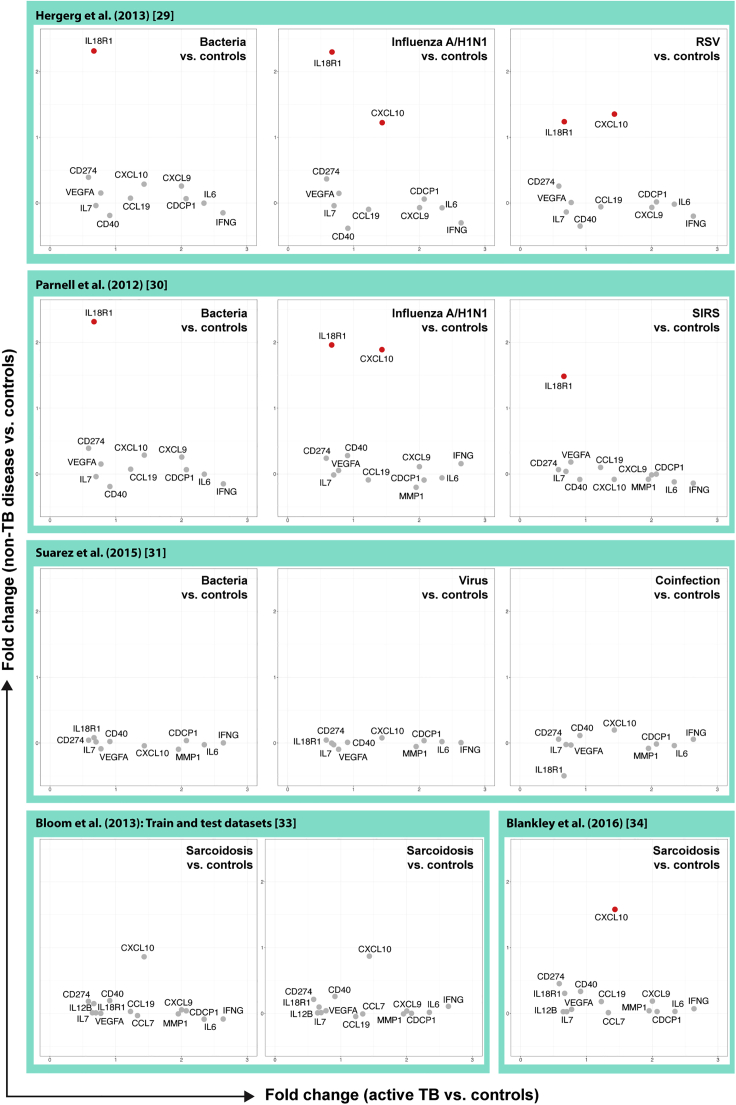
The expression of proteins of the turquoise module in our Swedish cohort compared with other viral/bacterial infections, SIRS, and sarcoidosis from different datasets The fold-changes of proteins of the turquoise module in our cohort (active TB versus healthy controls; x axis) compared with their fold-changes in other non-TB diseases (non-TB diseases versus healthy controls; y axis). Red points indicate proteins of the turquoise module; genes encoding them are significantly highly expressed in either other infections or other diseases (log2(FC) > 1 and p-value <0.05). (Row 1) Pediatric patients with severe respiratory tract infection: Bacterial: (n = 18, *Streptococcus pneumonia* (12), *Streptococcus pyogenes* (4), *Staphylococcus aureus* (2) including 5 with viral co-infection (non-influenza A H1N1/RSV); influenza A/H1N1 (n = 19), RSV (n = 22), HC (n = 33). (Row 2) Adult patients with severe CAP or SIRS requiring ICU-care: bacterial: (n = 16, mixed etiology) Influenza A/H1N1(n = 8), SIRS without infection (n = 12), HC (n = 18). (Row 3) Adult patients hospitalized for LRTI: Bacterial (n = 22; *S. pneumoniae* (13), *Moraxella catarrhalis* (4), *S. aureus* (2), mixed bacterial (3); viral (n = 71, Influenza A (32), RSV (17), influenza B (9), HMPV (7); mixed bacterial/viral (n = 25) HC (n = 40). (Row 4) Pulmonary sarcoidosis versus controls. RSV = respiratory syncytial virus; CAP = community-acquired pneumonia; ICU = intensive care unit; SIRS = systemic inflammatory response syndrome; LRTI = lower respiratory tract infection; HMPV = human metapneumovirus.

To investigate if the remaining 14 proteins were specific for active TB, we performed differential expression analysis using multiple transcriptomic datasets including either viral or bacterial lower respiratory tract infections (LRTI), systemic inflammatory response syndrome (SIRS), or sarcoidosis (Figure 3). This allowed us to identify genes encoding the signature proteins that were highly and significantly expressed (log2(FC) > 1 and p value <0.05) in diseases with clinical symptoms overlapping with active TB. To this end, we used 3 transcriptomic datasets, GEO: GSE42026,^24^ GSE40012,^25^ and GSE60244,^26^ each containing various types of lower respiratory infections. GSE42026 included (pediatric) patients with severe LRTI of different etiologies: bacterial (mostly *Streptococcus pneumoniae*), influenza A/H1N1/09, and respiratory syncytial virus (RSV) infection. GSE40012 included adult patients with severe community-acquired pneumonia (CAP), either bacterial or caused by influenza A/H1N1, and SIRS,^27^ without evidence of infection. Finally, GSE60244 included patients hospitalized for bacterial LRTI (*S. pneumoniae* being the most common), viral LRTI (influenza A, B, or RSV), and viral/bacterial co-infection. We also conducted the same experiment using 3 datasets from 2 studies containing sarcoidosis samples.^28^^,^^29^ Two genes, corresponding to the proteins IL18R1 and CXCL10 (IP-10), stood out in these analyses (Figure 3). IL18R1 was highly expressed in severe viral and bacterial LRTI and SIRS, whereas CXCL10 was highly expressed in severe viral LRTI and in one of the sarcoidosis studies. However, most of the proteins of the signature were only expressed at high levels in individuals with active TB. Therefore, in addition to LAP TGF-beta-1 and CSF-1 that were expressed only at very low levels, we also removed IL18R1 and CXCL10 from the signature, leading to a 12-marker plasma signature associated with active TB and low cross-expression to other bacterial/viral lower respiratory infections and sarcoidosis. The final signature consisted of the proteins interferon (IFN)-gamma, interleukin (IL)-6, CUB-domain containing protein 1 (CDCP1), CXCL9, matrix metalloproteinase (MMP)-1, monocyte chemotactic protein (MCP)-3, CCL19, CD40, vascular endothelial growth factor A (VEGFA), IL-7, IL-12B, and programmed cell death ligand 1 (PD-L1).

### Validating the 12-marker signature in TB and non-TB proteomic and transcriptomic datasets

**Figure 4:**
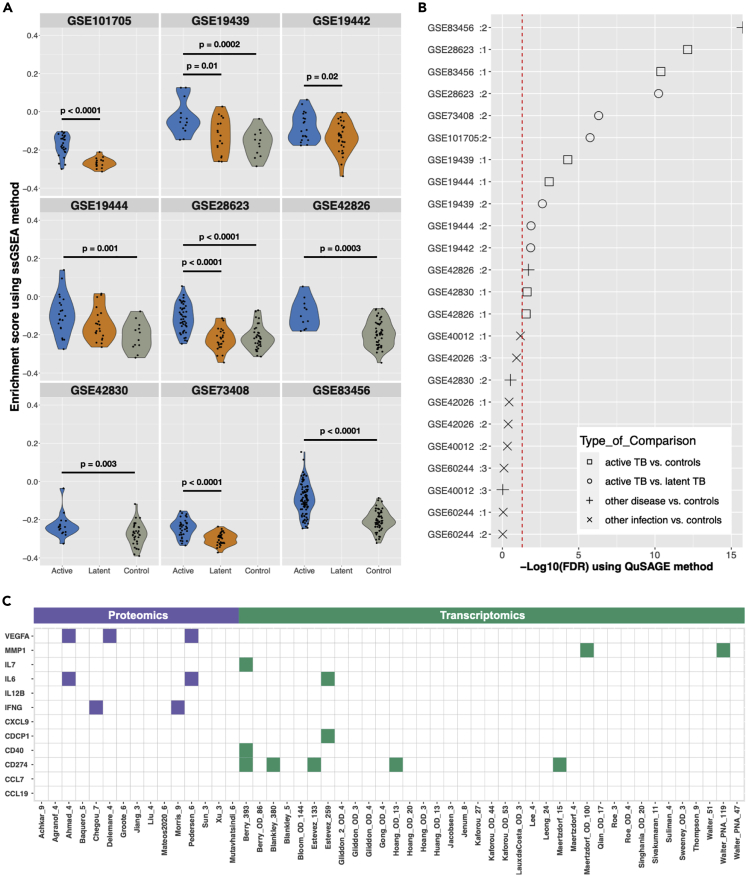
Validation and overlap of the 12-marker protein signature (A) ssGSEA indicating enrichment score for the 12-marker signature in different transcriptomic datasets. Blue indicates individuals with active TB, orange indicates latent TB, and gray indicates healthy controls. The p values show the statistical significance for the enrichment score difference between either active TB and latent TB or active TB and healthy controls using the Wilcoxon test. (B) QuSAGE analysis with –log(FDR-adjusted p value) indicating capacity of the 12-marker signature in separating active TB from healthy controls (open boxes), active TB from latent TB (open circles), non-infectious inflammatory disease from healthy controls (+), and other LRTIs or SIRS from healthy controls (×). The red line indicates FDR-adjusted p value <0.05. (C) Overlap between individual proteins in the 12-marker signature with other proteomic (left) and transcriptomic signatures (right) from the TBSignatureProfiler R package identifying active TB.

To assess the significance of the 12-marker signature in independent TB cohorts, we performed ssGSEA on published transcriptomic TB datasets^28^^,^^29^^,^^30^^,^^31^^,^^32^^,^^33^^,^^34^^,^^35^^,^^36^ from the curated TBData R package (Table S1). We observed higher enrichment scores in the active TB group, compared with either latent TB or healthy controls in all the transcriptomic TB datasets except one (GEO: GSE19444) (Figure 4A). We also used QuSAGE to compare the enrichment of the 12-marker signature in Mtb infections and other bacterial/viral infections and other pulmonary diseases (Figure 4B). Three different comparisons were done using the GEO: GSE42026,^24^ GSE40012,^25^ and GSE60244^26^ datasets to assess the signature in respiratory infections versus healthy controls and in sarcoidosis disease versus healthy controls using 3 transcriptomic datasets (GEO: GSE83456,^37^ GSE42826,^28^ and GSE42830^28^) (Figure 4B). We observed that the 12-marker signature was overrepresented in all comparisons between active TB and latent TB or healthy controls but was not significantly enriched in those gene sets comparing other LRTIs versus healthy controls. However, in 2 of the 3 sarcoidosis datasets the signature was also enriched, indicating that the signature might not, on its own, be able to distinguish active TB from sarcoidosis without also weighing in clinical data.

We compared the 12-marker signature with the other published gene signatures^12^^,^^28^^,^^29^^,^^32^^,^^33^^,^^34^^,^^38^^,^^39^^,^^40^^,^^41^^,^^42^^,^^43^^,^^44^^,^^45^^,^^46^^,^^47^^,^^48^^,^^49^^,^^50^^,^^51^^,^^52^^,^^53^^,^^54^^,^^55^^,^^56^ from the TBSignatureProfiler R package^36^ and published protein signatures^57^^,^^58^^,^^59^^,^^60^^,^^61^^,^^62^^,^^63^^,^^64^^,^^65^^,^^66^^,^^67^^,^^68^^,^^69^^,^^70^^,^^71^ to investigate the overlap with the proteins of our signature (Figure 4C). VEGFA, IL-6, and IFN-gamma were identified in at least 2 other proteomic studies,^58^^,^^59^^,^^60^^,^^61^^,^^62^ whereas CD274 (also called PD-L1) was observed in several published transcriptional signatures. IP-10 (also called CXCL10), which was removed due to its high expression in severe viral LRTs and sarcoidosis, appears in several proteomic studies.^59^^,^^60^^,^^61^^,^^62^ Although the other non-overlapping proteins might not have been included in protein signatures before, they have been associated with TB^72^^,^^73^^,^^74^^,^^75^ and could potentially be stimulated/produced via similar signaling pathways in response to mycobacterial infection, such as has been indicated for signal transducer and activator of transcription 1 (STAT1) in TB.^76^ To assess if this was the case, we used the StrongestPath application^77^ in Cytoscape to evaluate how the proteins were connected to different STAT transcription factors based on data from the KEGG database (Figure S10). We observed that several of the proteins were directly associated with STAT1 (PD-L1, IFN-gamma, VEGFA, CXCL9, and IL-12B), consistent with previous literature,^76^ to a lesser extent with STAT3 (PD-L1, VEGA, IL-12B) and STAT4 (IFN-gamma), and indirectly with STAT2 and STAT5A (PD-L1, IFN-gamma, VEGFA, CXCL9, IL-12B, IL-6, and MMP-1).

### Association between the 12-marker signature and disease severity

**Figure 5:**
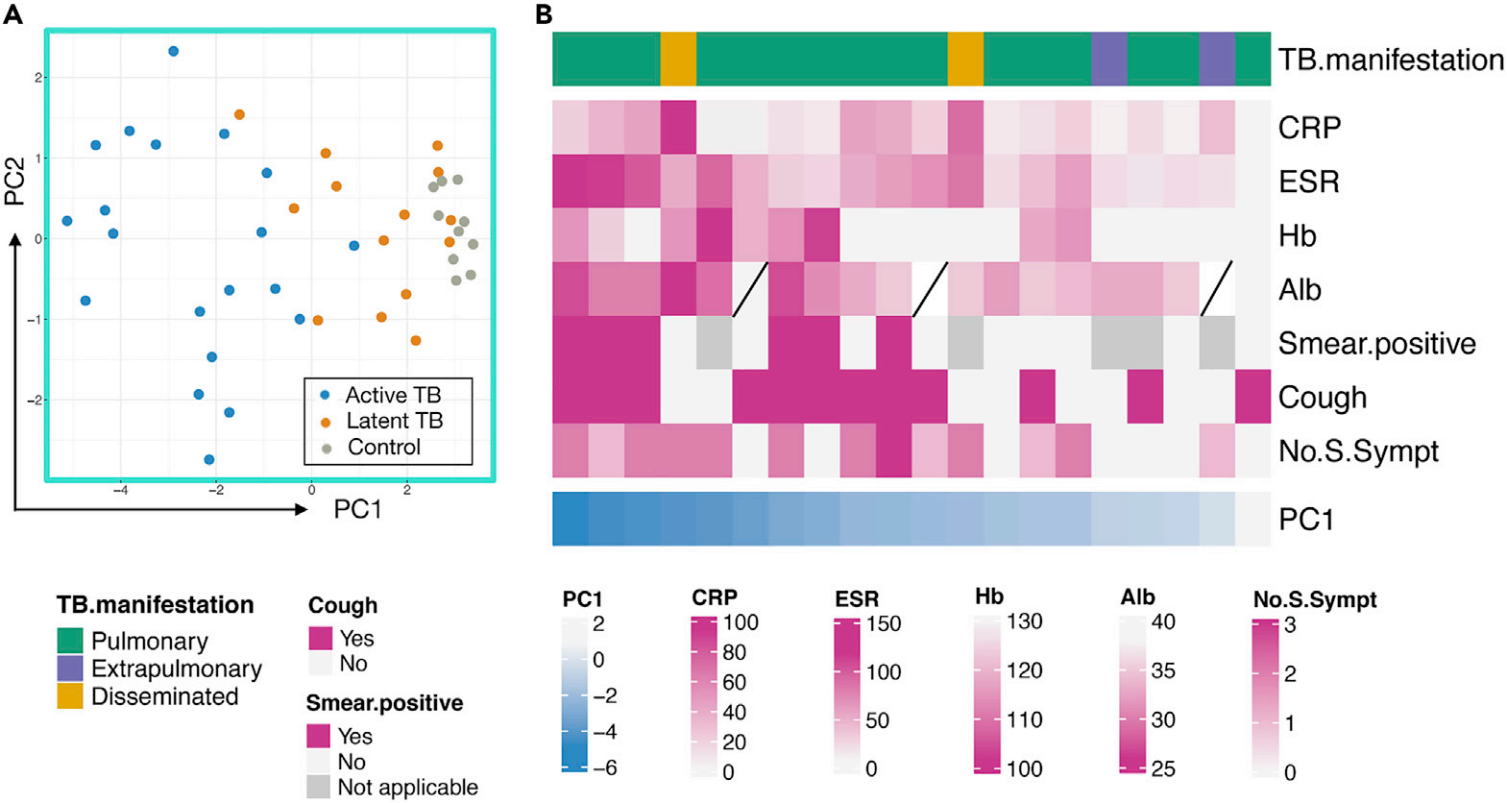
Association between the first principal component (PC1) and clinical characteristics of active TB (A) PC1 and PC2 indicate variation in concentrations of the 12-marker signature for individuals with active TB (blue dots), latent TB (orange dots), and healthy controls (gray dots). (B) Clinical characteristics (rows) of individual active TB donors (columns) stratified by the negative value of PC1 (the more to the left indicating a stronger signature). TB manifestation: pulmonary (including 3 with pleuritis); 2 disseminated (pulmonary/abdominal/lymphnode and liver/lymphnode) and 2 extrapulmonary (lymphnode and soft tissue/osteitis), CRP = C-reactive protein, ESR = erythrocyte sedimentation rate, Hb = hemoglobin, Alb = albumin. Smear-positive; sputum smear microscopy positive (5 of 14 with sputum); cough: as reported by patient; presence of systemic symptoms (fever, weight loss, night sweats); no.S.Sympt = number of systemic symptoms; night sweats, fever, and weight loss.

Principal component analysis of the 12-marker signature (Figure 5A) showed that similar to the turquoise module (Figure S5), active TB was primarily separated from latent TB and healthy controls by principal component 1 (PC1). We, therefore hypothesized that PC1 could represent a proxy for the magnitude of the inflammatory response and disease severity. To evaluate if this was the case, we stratified the individuals with active TB based on their PC1 values and a set of clinical features (Figure 5B, Table S2). The heatmap shows that samples with a smaller PC1 value, located toward the left side of the heatmap, were associated with several biochemical markers indicating more extensive inflammation, such as lower hemoglobin (Hb) and albumin (Alb), and higher erythrocyte sedimentation rate (ESR) and C-reactive protein (CRP). The presence of any systemic symptoms (fever, night sweats, and weight loss) and smear positivity was also associated with a lower PC1 value. We next performed a regression analysis to investigate if individual or combinations of the clinical variables could explain the PC1 values. A standard least squares model including ESR (estimate: −0.028, 95% confidence interval [CI]: −0.016 to −0.041, p = 0.0005) and Alb (estimate: 0.20, 95% CI: 0.08–0.33, p = 0.006) was highly associated with PC1 (_adj_r^2^ = 0.74, p < 0.0001), clearly associating the signature with clinical presentation.^78^

The 3 active TB cases with the weakest PC1 signal had limited symptoms and inflammation (Figure 5B). One patient had a culture-confirmed lymph node TB. Another patient had PCR-positive but culture-negative lymph node and pulmonary TB. The active TB case with the lowest inflammatory signal was found through contact investigation very early in active disease progression, with cough as the only symptom but with no inflammation in standard laboratory tests. This suggests that the signature has the potential to identify active TB very early on in disease progression.

Although the signature was not optimized for the detection of latent TB, the visually apparent separation of latent TB from healthy controls (Figure 5A) could possibly indicate a TB-specific immune response detectable in the plasma of these individuals. Two of the 3 latent TB cases clustering with the healthy controls had no recent exposure to TB and were clinically classified as remotely acquired infections (i.e., no known exposure to a TB case for >2 years). In summary, these data suggest that the signature could be helpful in identifying TB disease progression or cure.

## Discussion

In this exploratory study, we identified a signature of 12 co-expressed proteins that are highly enriched in individuals with active TB compared with latent TB and healthy controls. The signature includes the proteins IFN-gamma, IL-6, CDCP1, CXCL9, MMP-1, MCP-3, CCL19, CD40, VEGFA, IL-7, IL-12B (subunit p40), and PD-L1.

IFN-gamma and IL-12 are well described as important mediators of Mtb control, and their levels increase in plasma during active TB.^79^^,^^80^ IL-6 and IL-7 have also been shown to be increased in active TB,^81^^,^^82^ where elevated IL-6 levels were associated with cavitary TB.^81^ The chemokine ligands CXCL9, MCP-3 (CCL7), and CCL19 are associated with the recruitment of dendritic cells, T cells, and natural killer (NK) cells (MCP-3) to the lung (CXCL9) and lymph nodes (CCL19) in TB.^83^ CXCL9 and MCP-3 have been shown to increase in blood^73^^,^^84^ in TB patients, whereas CCL19 has primarily been associated with granuloma formation and maintenance in mouse models of TB.^75^ Matrix metalloproteinases, such as MMP-1, have been suggested to have an important role in tissue remodeling in TB.^85^ In support of this, MMP-1 is selectively upregulated and secreted during TB,^86^ consistent with MMP-1 expression being regulated via nuclear factor κB (NF-κB) activation, and modulated via IFN-gamma, IL-6, and IL-12.^35^ VEGFA is also associated with tissue remodeling, as it promotes blood vessel formation. It can be produced by a subset of macrophages in Mtb granulomas in mice.^87^ In human and rabbit granulomas, VEGFA induces an abnormal and dysfunctional vasculature, which reduces the delivery of low-molecular-weight molecules,^72^ potentially affecting chemoprophylactic drug perfusion. CD40, PD-L1 (CD274), and CDCP1 (CD318) are primarily described in their role as cell surface receptors/ligands. CD40 is expressed on antigen-presenting cells (APCs), whereas its ligand CD40L is mainly expressed by activated T cells. The CD40–CD40L interaction provides an activating signal in both APC and T cells, leading to, e.g., increased production of IFN-gamma.^88^ The levels of CD40L are commonly reduced on peripheral blood cells in TB patients,^89^ but the potential role of elevated circulating CD40 remains largely unexplored. PD-L1 is similarly expressed by APCs and binds to its receptor PD-1, which is expressed at high levels on exhausted T cells in TB.^90^ The receptor−ligand interaction contrasts with CD40−CD40L by primarily inhibiting T cell function.^90^^,^^91^ Soluble PD-L1 has been highlighted as a biomarker for several different types of cancer and can function as a decoy for immunotherapy,^92^ but it remains unclear if it can interact with PD1 on cells and/or if that would lead to biological effects. CDCP1 is less well described. It is normally expressed on the surface of epithelial cells and certain stem cells where it can act as a ligand for CD6 to activate T cells.^93^ It is via this interaction associated with certain autoimune diseases, but there is not yet a role described in TB.

Although the proteins included in the 12-marker signature are all correlated with each other, it remains unclear if they are co-regulated or respond to the infection via different pathways. As described earlier, some of the proteins are regulated via activation of the same STATs, which could influence their joint expression and is consistent with, for example, STAT1 being an important immune response pathway in Mtb infection.^76^ On an individual patient level, the signature was associated with disease activity with a stronger protein signature significantly related to perturbations in common clinical inflammatory markers (ESR and albumin) and reported symptoms. This is consistent with studies where a stronger inflammatory response is associated with higher mycobacterial burden and more lung cavitation.^80^^,^^81^

When measuring inflammatory mediators to be used as biomarkers for a given disease in the absence of disease-specific stimulation, it is important to assess the specificity of the biomarkers in diseases with a similar clinical presentation. In this study, we show that the identified signature was differentially expressed in independent TB transcriptomic datasets and importantly, that it is not expressed in patients with other lower respiratory tract infections. However, when testing the signature in a sarcoidosis dataset,^29^ we observed a significant enrichment of the proteins. Sarcoidosis and TB have previously been shown to have similar gene expression patterns.^29^ Both diseases demonstrate an interferon-driven gene upregulation, although as shown by Blankley et al., on a group level this pattern is more strongly expressed in active TB, reflecting disease activity.^29^ We were not able to directly compare sarcoidosis versus active TB to assess if the signal is higher in one group or the other. However, if the signature was to be used as a screening test, this potential overlap will likely not pose a significant diagnostic problem in a clinical setting, as symptoms and other clinical information such as radiology will help separate the two conditions. Further, in TB high-endemic countries where a screening test is most urgently needed, the prevalence of sarcoidosis is very low compared with pulmonary TB.^94^^,^^95^

Lack of overlap between protein signatures for active TB has been previously described, and the methods used to quantify proteins in different studies also vary.^14^ When comparing the 12-marker signature with recently published protein signatures^58^^,^^59^^,^^60^^,^^61^^,^^62^^,^^63^^,^^64^^,^^65^^,^^66^^,^^67^^,^^68^^,^^69^^,^^70^^,^^78^^,^^96^ some biomarkers reappear, although the overlap is limited. In addition, in the published proteomic studies, there were none with processed data that included all the proteins of our signature in their dataset, making us unable to use them to validate the signature proposed in this study.

VEGF and IL-6, present in the 12-protein signature, together with IL-8 and IL-18, constitute a 4-protein signature identified by Ahmad et al.^58^ They analyzed 47 proteins with Luminex, and the 4 selected proteins were validated in serum from three different patient cohorts collected through the FIND initiative. The sensitivity for active TB in TB suspects was 80% (95% CI, 73%–85%), and the specificity was 65% (95% CI, 57%–71%). Interestingly, there was quite a large overlap between the Olink inflammation panel we used and their 47-protein Luminex panel. Although IL-8 and IL-18 were more abundant in active TB patients compared with healthy controls in our dataset (Figure S4), the fold-change values were low compared with the proteins included in the 12-protein signature and were not significantly higher when compared with latent TB individuals.

VEGF was also included in a 4-protein signature (CCL1, CXCL10, ADA-2, VEGF) proposed by Delemarre et al.,^59^ where they compared active TB with treated and untreated latent TB. The signature was validated in two separate patient cohorts with a sensitivity of 95% and a specificity of 90%. CXCL10 (IP-10) was also highly expressed in our analysis but was removed due to its high expression in other lower respiratory tract infections. CCL1 and ADA-2 were not analyzed in the current study.

Although VEGF was not included in the 7-protein signature described by Chegou et al.,^60^ it was increased in active TB patients in their cohort. Of their 7 proteins, IFN-gamma overlaps with our 12-protein signature. CXCL10 again appears in their signature, whereas the other proteins (CRP, TTR, CFH, APO-A1, SSA) were not part of the proteins evaluated in the current study.

In 2021, Morris et al.^61^ and Mutavhatsindi et al.^70^ investigated the same 22 proteins as Chegou et al.^60^ and attempted to validate the 7-protein signature in patients with suspected TB. In the first study, the sensitivity was very high (98%), whereas the specificity was low (12%). They argued that this might be due to the different patient cohorts—primary care level versus hospital care level. Instead, they identified a 9-protein signature (fibrinogen, alpha-2-macroglobulin, CRP, MMP-9, transthyretin, complement factor H [CFH], IFN-gamma, IP-10, and TNF-alpha) where IFN-gamma and IP-10 reappear. In the test set, the sensitivity was 92% (95% CI, 80%–98%) and specificity 71% (95% CI, 56%–84%) for diagnosing culture-verified TB from other diseases in TB suspects. Their second study^70^ failed to validate the 7-marker signature. Instead, they proposed CRP and CCL1 as a signature that performed equally well in both HIV^−^ and HIV^+^ individuals. Trying to design a protein signature based on previous findings, Pedersen et al.^62^ evaluated 9 proteins in pulmonary TB patients compared with healthy controls. They found that IL-6, VEGFA, (and IP-10) were increased in active TB. Their proposed signature consists of IP-10 and 4 miRNA molecules, including miR-29a, miR-146a, miR-99b, and miR-221. IP-10 is also part of the 5-protein signature identified by Luo et al.^97^

Garay-Baquero et al. analyzed more than 5000 peptides using mass spectrometry on a relatively small discovery cohort (10 individuals with active TB and 10 healthy controls).^64^ They identified 46 proteins to be overexpressed in active TB and selected 9 and 7 proteins for validation in larger populations in South Africa and the UK, respectively. They used Luminex or ELISA and compared active TB with other respiratory diseases (ORD) and healthy controls. The proposed 5-protein signature—CFHR5, LRG1, CRP, LBP, and SAA1—performed well with an AUC >0.8 in both settings for active TB vs ORD. There was no overlap with the 12-protein signature identified here, and none of the proteins were among the 136 proteins identified as associated with active TB (46 more abundant and 90 less abundant) in their discovery phase, although the use of different methods makes direct comparison difficult.

Another large proteomic study by de Groote et al.^71^ used SOMAscan to measure over 4,000 proteins in 1,470 patient samples from pulmonary TB patients and other TB suspect cases and resulted in a 6-protein signature. Although their signature did not overlap with our 12-protein signature, IL-6 and MMP-1 were overexpressed in their active TB cases.

In summary, IFN-gamma, IL-6, and VEGF, together with IP-10, were identified as markers for active TB in several previous proteomic studies, as well as identified in our study. These are all regulated by STAT-1, which has previously been identified as an important immune response pathway in Mtb infection^76^ and potentially explains why these proteins are identified in different studies. However, as found here, IP-10 is also highly expressed in other severe infectious diseases and as such is likely not specific for TB in the absence of Mtb-specific stimulation.^98^

### Limitations of the study

This study is based on relatively few individuals. Although a small sample cohort does not preclude the possibility of identifying effective biomarkers, it is possible that the study population becomes too homogeneous and that the biomarkers identified will not work in a different sample population. Albeit small, this study cohort is well defined, with microbiological verification of all active TB cases, a wide range of disease severity, and with large variation in patient origin, reflecting the TB population in Sweden and thus increasing the likelihood of generalizability to other geographic regions. In the absence of protein datasets for validation, we used nine transcriptomic datasets including over 3,000 individuals from 4 continents to assess if the signature was enriched among active TB patients compared with other diseases. A major caveat with this type of validation is the relatively poor correlation between mRNA and protein quantities.^99^ For this reason, we did not perform further optimization and assessment of the sensitivity and specificity of the signature in the transcriptomic datasets, but rather only assessed overall enrichment. For further optimization and validation studies, independent protein datasets will be important. Although the Olink PEA has been used increasingly used in scientific studies, it is not yet available for clinical practice. The method also has a relatively frequent sample abort rate, as seen in this study, with 3 out of 47 samples not passing assay quality control. It will therefore also be important to further refine the signature and adapt to established clinical assay platforms.

## STAR★Methods

### Key resources table

**Table:** 

REAGENT or RESOURCE	SOURCE	IDENTIFIER
**Biological samples**		

Sodium heparin plasma from a Swedish TB cohort	Karolinska University Hospital Biobank	BbK1792

**Critical commercial assays**		

Target 96 Inflammation panel	Olink Proteomics AB	Cat#95302

**Software and algorithms**		

R 4.1.1	R Core Team, 2019	https://www.r-project.org/
OlinkAnalyze (R package)	N/A	https://github.com/Olink-Proteomics/OlinkRPackage
Limma (R package)	(Ritchie et al., 2015)^100^	https://bioconductor.org/packages/release/bioc/html/limma.html
WGCNA (R package)	(Langfelder et al., 2008)^101^	https://horvath.genetics.ucla.edu/html/CoexpressionNetwork/Rpackages/WGCNA/
ssGSEA (R package)	(Yi et al., 2020)^104^	https://github.com/broadinstitute/ssGSEA2.0
GSVA (R package)	(Hänzelmann et al., 2013)^102^	https://github.com/rcastelo/GSVA
qusage (R package)	(Yaari et al., 2013)^103^	https://bioconductor.riken.jp/packages/3.9/bioc/html/qusage.html
curatedTBData (R package)	(Wang et al., 2021)^23^	https://github.com/compbiomed/curatedTBData
TBSignatureProfiler (R package)	(Johnson et al., 2021)^23^	https://github.com/compbiomed/TBSignatureProfiler
ggplot2 (R package)	N/A	https://cran.r-project.org/web/packages/ggplot2/index.html
ComplexHeatmap (R package)	(Gu et al., 2016)^107^	https://github.com/jokergoo/ComplexHeatmap
circlize (R package)	(Gu et al., 2014)^106^	https://github.com/jokergoo/circlize
Cytoscape 3.9.1	(Shannon et al., 2003)^22^	https://cytoscape.org/
StrongestPath	(Mousavian et al., 2021)^77^	https://apps.cytoscape.org/apps/strongestpath

### Resource availability

#### Lead contact

Further information and requests for resources and reagents should be directed to and will be fulfilled by the Lead Contact, Christopher Sundling (christopher.sundling@ki.se).

#### Materials availability

This study did not generate new unique reagents.

### Experimental model and subject details

#### Ethical considerations

The study was registered and granted ethical permission from the Swedish Ethical Review Board, EPN-number 2013/1347–3½ and 2017/2262–32. All study subjects were adults and signed written informed consent forms after receiving written and verbal information, in relevant cases using professional interpreter services.

#### Study design

The study was designed as an experimental data-driven exploratory study with the aim to measure inflammatory proteins in the plasma of individuals with active TB and compare to individuals with latent TB and healthy controls to identify a biomarker signature associated with active TB.

#### Study participants

Adult patients (>18 years old) with active and latent TB were recruited randomly from patients seen at the TB department at the Karolinska University Hospital, Stockholm from May 2018 to November 2019. Eligible for the study were: 1) Patients with active TB sampled within 1 week (one patient was 9 days) of anti-TB treatment initiation. Active TB was defined through microbiological verification via Mtb culture, or if culture negative, other microbiological positive results for Mtb (microscopy or PCR) combined with clinical and radiological findings and response to anti-TB treatment (active TB individuals are further described in Table S2); 2) Individuals with latent TB that had a positive IGRA result (QuantiFERON-TB Gold In-Tube [QFT-GIT] or QuantiFERON-TB Gold Plus [QFT-Plus]) identified through contact investigation or screening of migrants, for which active TB had been excluded. The cut-off between recent and remote latent TB was set to two years after exposure. For healthy controls, adult students and staff without previously known TB exposure, co-morbidities, or immunosuppression were invited. The gender distribution was similar between the groups, and the age was similar between active and latent TB, but overall younger for healthy controls.

#### Data collection

Demographic, epidemiological, and clinical data for patients with active and latent TB were extracted from patient charts. For all subjects this included information regarding previous exposure to patients with active TB, co-morbidities, current medication, and radiological, biochemical, and microbiological test results. For the active TB cases, clinical symptoms of TB disease were noted, and patients were classified according to pulmonary or extrapulmonary TB. Microbiological samples for mycobacterial analysis were collected independent of the study in accordance with clinical practice. All patient samples were analyzed at Karolinska University Hospital Laboratory with smear fluorescence microscopy (BX41, Olympus) for detection of acid-fast bacilli, PCR for detection of Mtb complex and molecular markers of resistance to isoniazid (*inhA, katG*) and/or rifampicin (*rpoB*) (BD MAX^TM^ Systems), as well as mycobacterial culture in liquid (BACTEC™ MGIT™ 960) and solid (Löwenstein-Jensen) medium. Drug susceptibility testing (DST) was routinely performed in an automated detection system (BACTEC^TM^ MGIT^TM^ 960 SIRE/PZA).

### Method details

#### Analysis of plasma proteins by proximity extension assay

Venous blood samples were collected in heparin vacutainer tubes and transferred to the laboratory for immediate preparation. The tubes were centrifuged at 670 *g* for 8 min after which the plasma fraction was aliquoted and stored at −80°C.

Patient plasma was analyzed using a proximity extension assay, a qPCR technology that simultaneously measures combinations of cytokines in preselected panels.^16^ We used the Olink® Target 96 inflammation panel, analysing 92 protein biomarkers (Table S3). In this automated process, specific antibodies carrying single-stranded matching DNA bind in pairs to each of the target proteins allowing for DNA hybridization and subsequent DNA-extension. The resulting DNA, unique for each target protein, is then subject to PCR amplification and finally detection. The generated results consist of normalized protein expression (NPX) values that correspond to log2 transformed relative protein concentrations. The assay was performed by the Translational Plasma Profile Facility at SciLifeLab, Stockholm, Sweden.

### Quantification and statistical analysis

#### Protein expression data analysis

Repeated samples were used between experimental batches for running bridge normalization. We used *read_NPX* and *olink_normalization* functions from the OlinkAnalyze R package (https://github.com/Olink-Proteomics/OlinkRPackage) to read the log2 NPX protein expression values and perform bridge normalization between batches of our data respectively. Proteins with NPX values below the limit of detection (LOD) in more than 30% of samples were filtered out. To remove batch effects from the final dataset, we applied the *removeBatchEffect* function from the Limma R package^100^ (Figure S11). We also performed differential expression analysis using the Limma R package.

#### Weighted co-expression network analysis

We used the WGCNA R package^101^ to construct a weighted protein co-expression network among the proteins of our dataset. *Pearson correlation* and the *signedhybrid* network type were used in the *adjacency* function of the WGCNA package. In the *signedhybrid* network type, only links associated with positive correlations were retained in the network, and negative correlations were discarded. Since most of the biological networks have a scale-free topology, the WGCNA package in the *pickSoftThreshold* function tries to find the best value of the power parameter to make a scale-free network. After network construction, the network was clustered into modules containing proteins that were highly positively co-expressed using a hierarchical clustering algorithm as implemented in the *cutreeDynamic* function of the WGCNA package with parameters deepSplit = 4 and minClusterSize = 5, and other parameters set as default. The expression profiles of proteins in each module were summarized by module eigengenes. In the *moduleEigengenes* function from the WGCNA package, the first principal component of the expression data of each module is measured as a module eigengene for that module. To find a module associated with TB progression, the correlation between each module eigengene and the trait vector was computed to identify which module that had a significant correlation with TB progression.

#### Protein signature enrichment analysis

To further validate the protein signature obtained from the co-expression network analysis, we applied two computational methods (ssGSEA^104^ and QuSAGE^103^) for enrichment analysis in independent transcriptomic datasets. In ssGSEA, the enrichment scores of the protein signature were calculated per sample based on the absolute value of proteins expression in that sample to quantify how much the protein signature was overrepresented in a specific sample. Moreover, to verify that the protein signature was specific to active TB and not to other non-TB respiratory infections or diseases with clinical presentations similar to active TB, the *qusage* function from the QuSAGE R package was applied to datasets comparing active TB or non-TB disease to various control groups. The gene set differential expression was calculated by combining individual probability functions obtained from individual differential expression of genes in a particular comparison. A p value, calculated by QuSAGE, determined the statistical significance of a gene set for each given comparison. FDR correction was applied to correct for multiple comparisons and a p < 0.05 was considered significant. We also used different types of single sample gene set enrichment analysis algorithms, including ssGSEA, GSVA^102^ and *Z* score, ^105^ all implemented in the gsva R package, to compare all co-expressed modules in terms of enrichment in active TB versus latent TB and healthy controls.

## Data Availability

Plasma protein data reported in this paper will be shared by the lead contact upon request. All code used in the analyses is deposited on https://github.com/SundlingLab/Olink_ATBsignature. Any additional information required to reanalyze the data reported in this paper is available from the lead contact upon request.
