# Supplementary material for: A protein signature associated with active tuberculosis identified by plasma profiling and network-based analysis
Source: iScience. 2022 Nov 22;25(12):105652. doi: 10.1016/j.isci.2022.105652 (PMC9763869; doi:10.1016/j.isci.2022.105652)
Supplement: Document S1. Figures S1–S11 and Tables S1–S3 [file mmc1.pdf]

iScience, Volume *25*

Supplemental information

A protein signature associated with active tuberculosis identified by plasma profiling and network-based analysis

Zaynab Mousavian, Elin Folkesson, Gabrielle Fröberg, Fariba Foroogh, Margarida Correia-Neves, Judith Bruchfeld, Gunilla Källenius, and Christopher Sundling

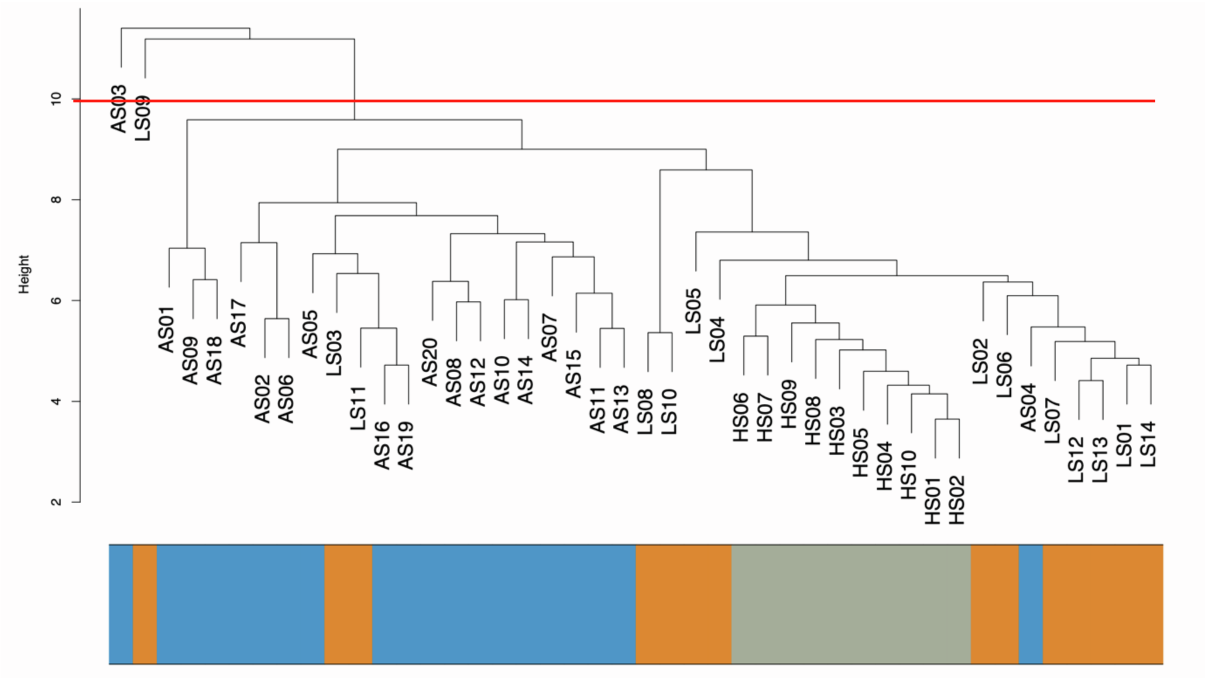

Figure S1. Hierarchical sample clustering to detect outliers. Two samples, one from the active TB group and one from the latent TB group were identified as outliers and removed prior to co-expression analysis, Related to Table 1.

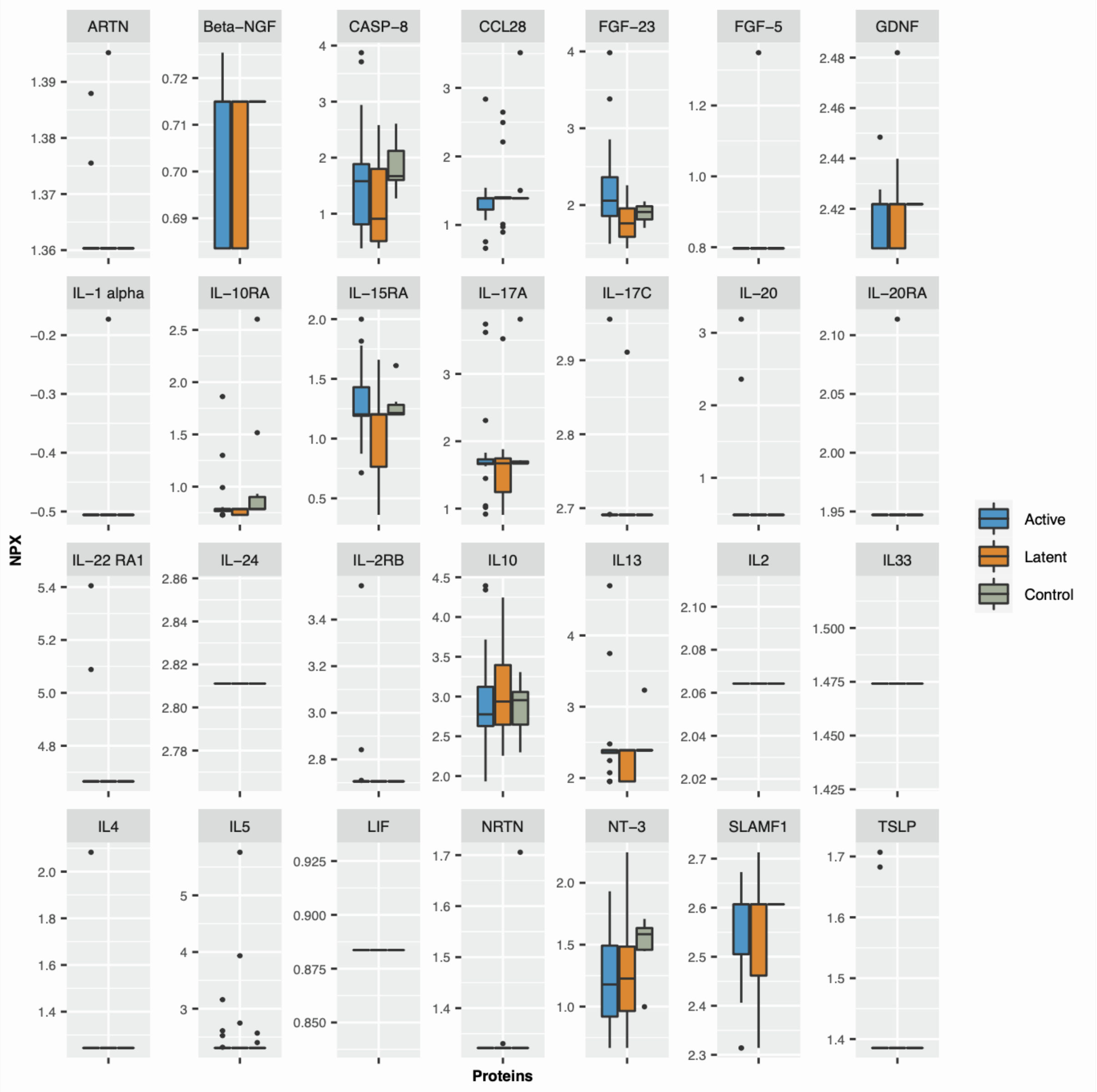

Figure S2. The NPX values of removed proteins in different groups of individuals, Related to STAR Methods.

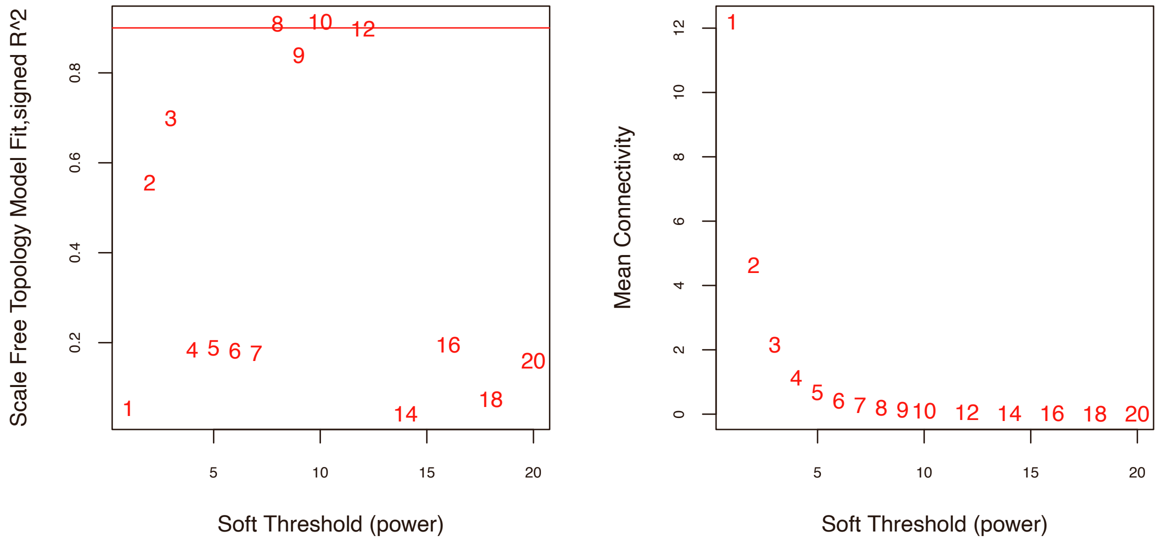

Figure S3. The power parameter for the Scale-free topology, Related to STAR Methods.

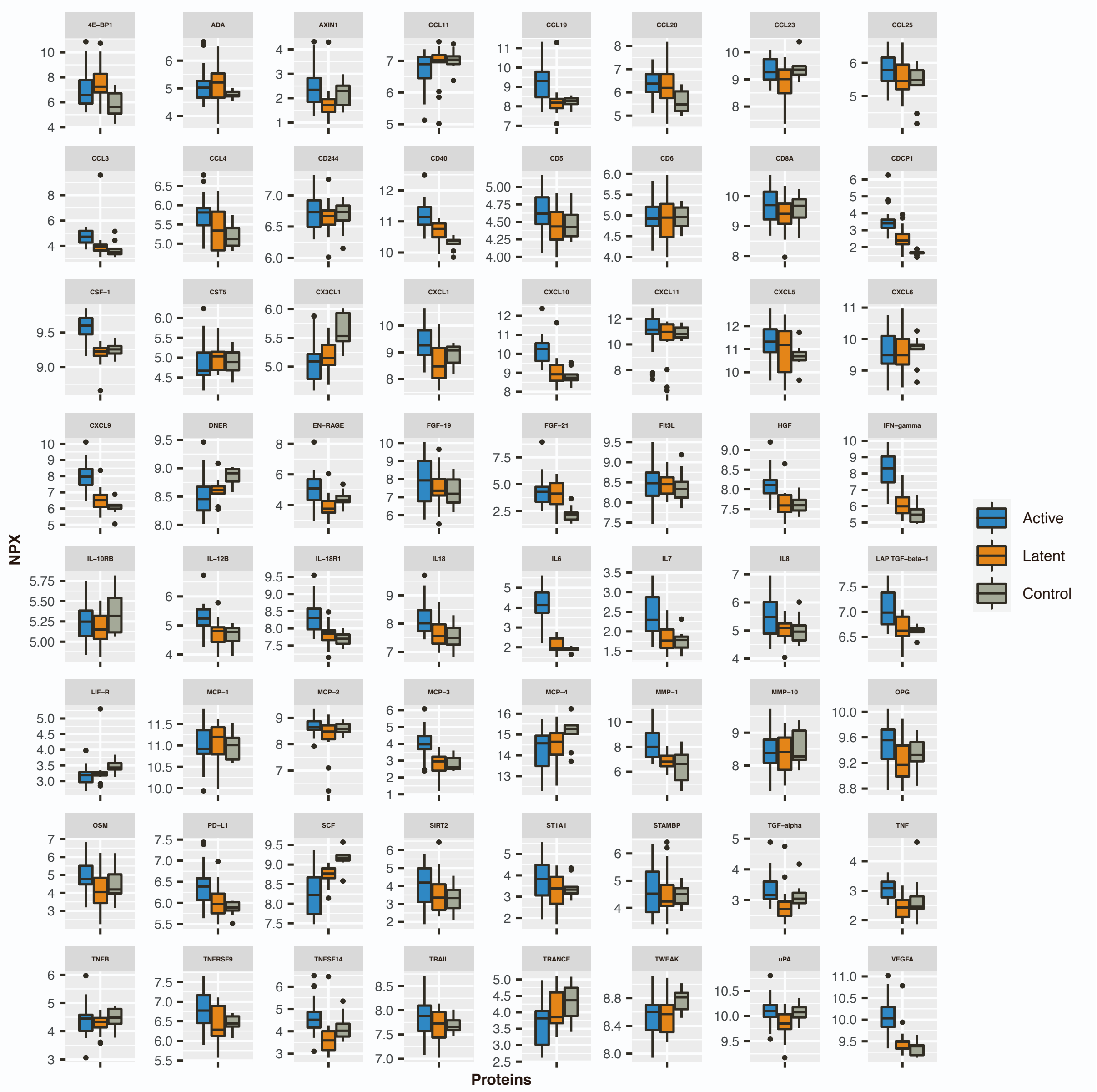

Figure S4. The NPX values of all remaining proteins in different groups of individuals, Related to STAR methods.

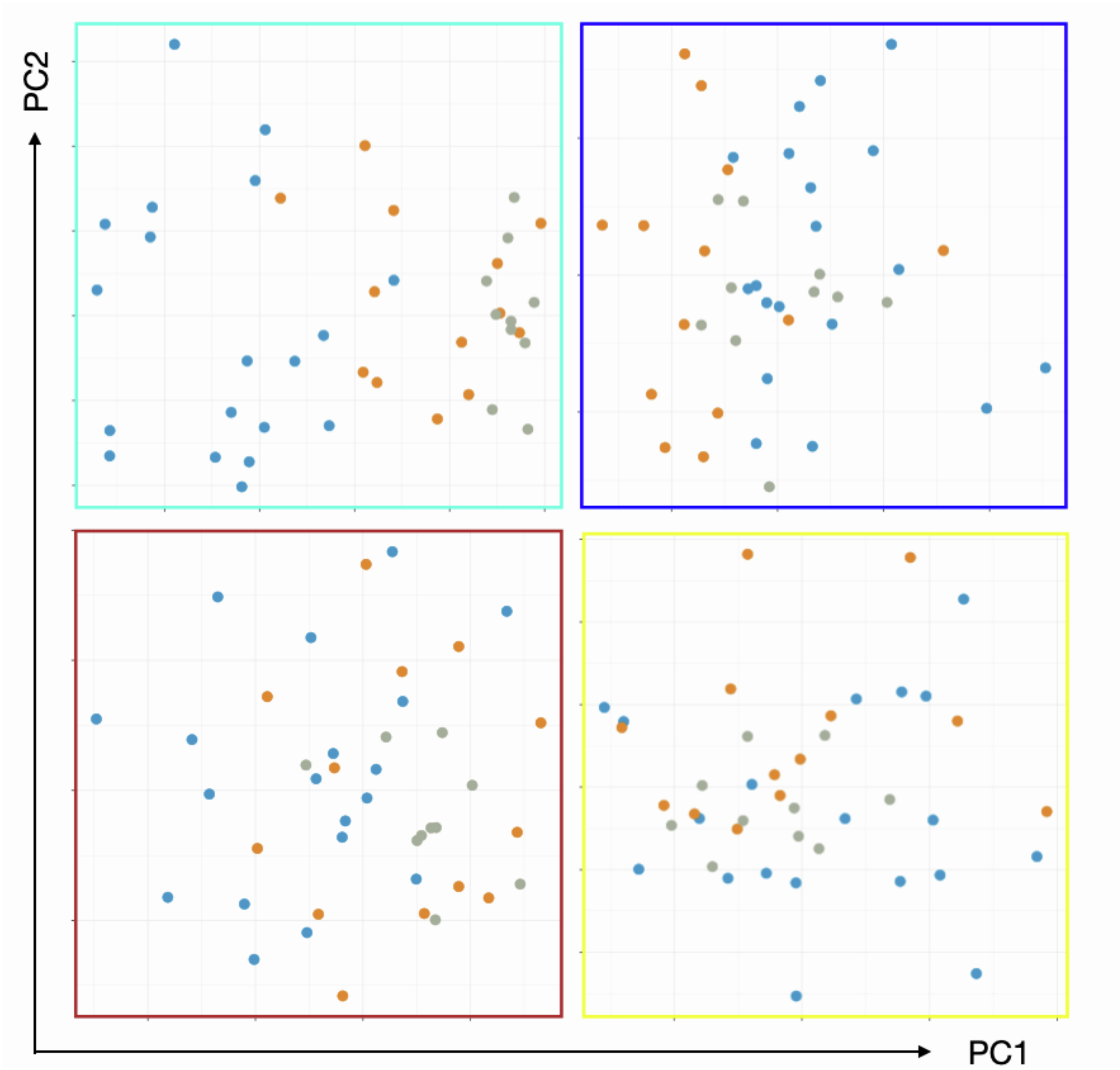

Figure S5. Visualizing samples regarding PC1 and PC2, obtained from the expression data of each module. Modules are surrounded indicated by colours including turquoise, blue, brown and yellow. Each point in plots represents one sample from one out of three groups of active TB (blue), latent TB (orange) and healthy control (grey), Related to Figure 1.

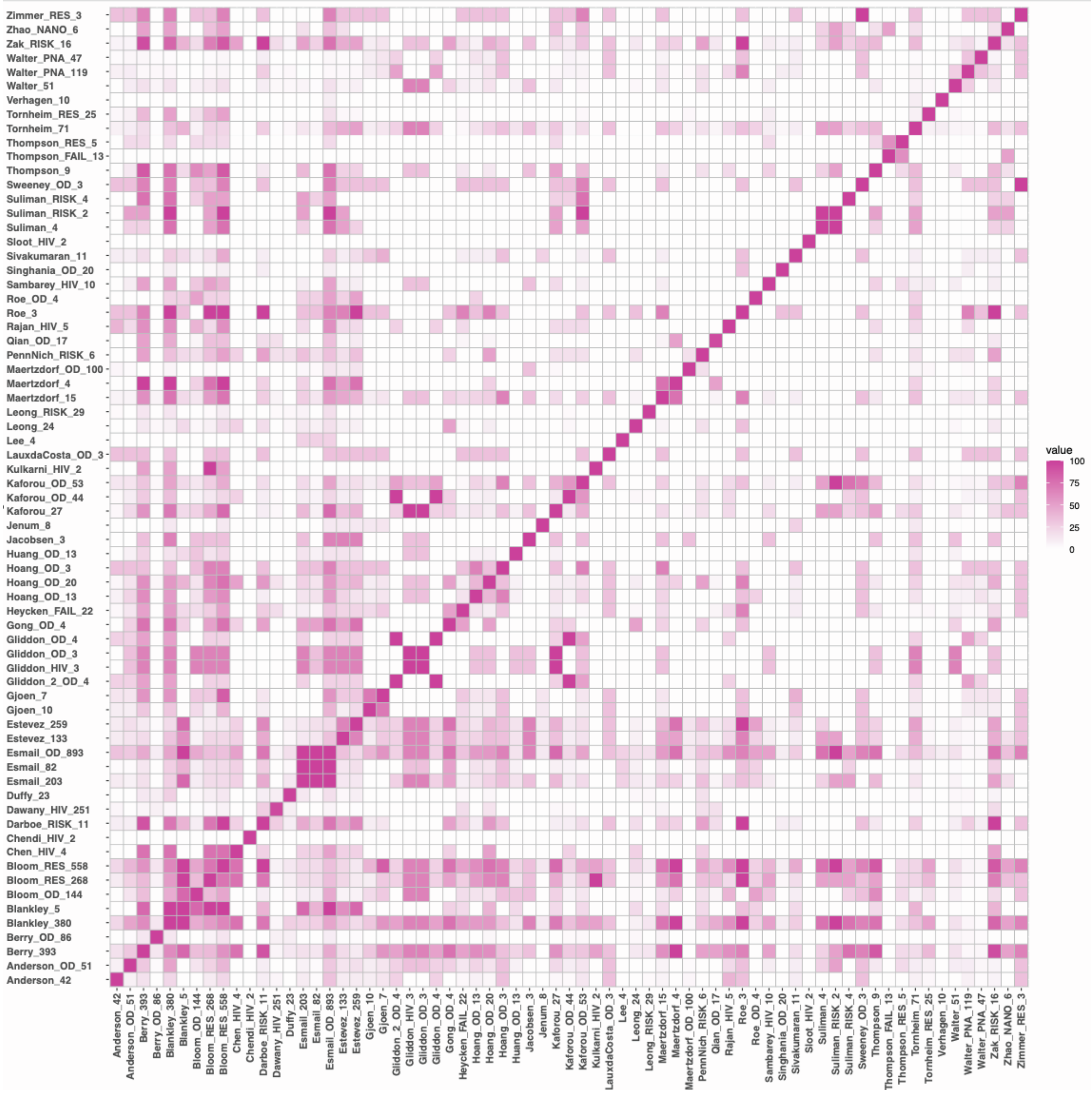

Figure S6. The overlap between the other published gene signatures from the TBSignatureProfiler R package. The value in each cell matrix indicates the percentage of genes that are shared between two distinct gene signatures, Related to Figure 4C.

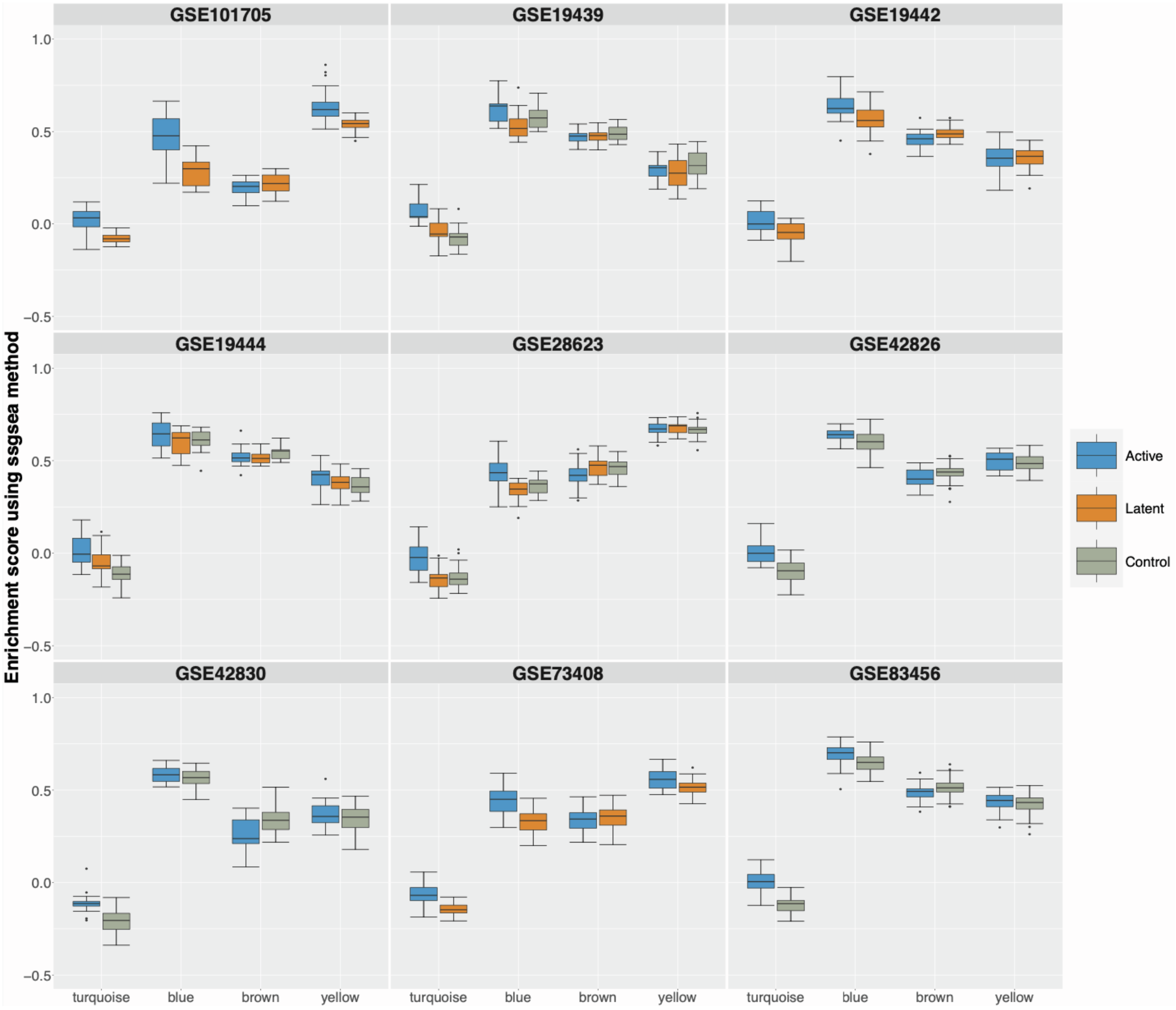

Figure S7. The enrichment analysis of all modules (from left to right: turquoise, blue, brown and yellow) on different transcriptomic datasets using the ssgsea method, Related to STAR Methods.

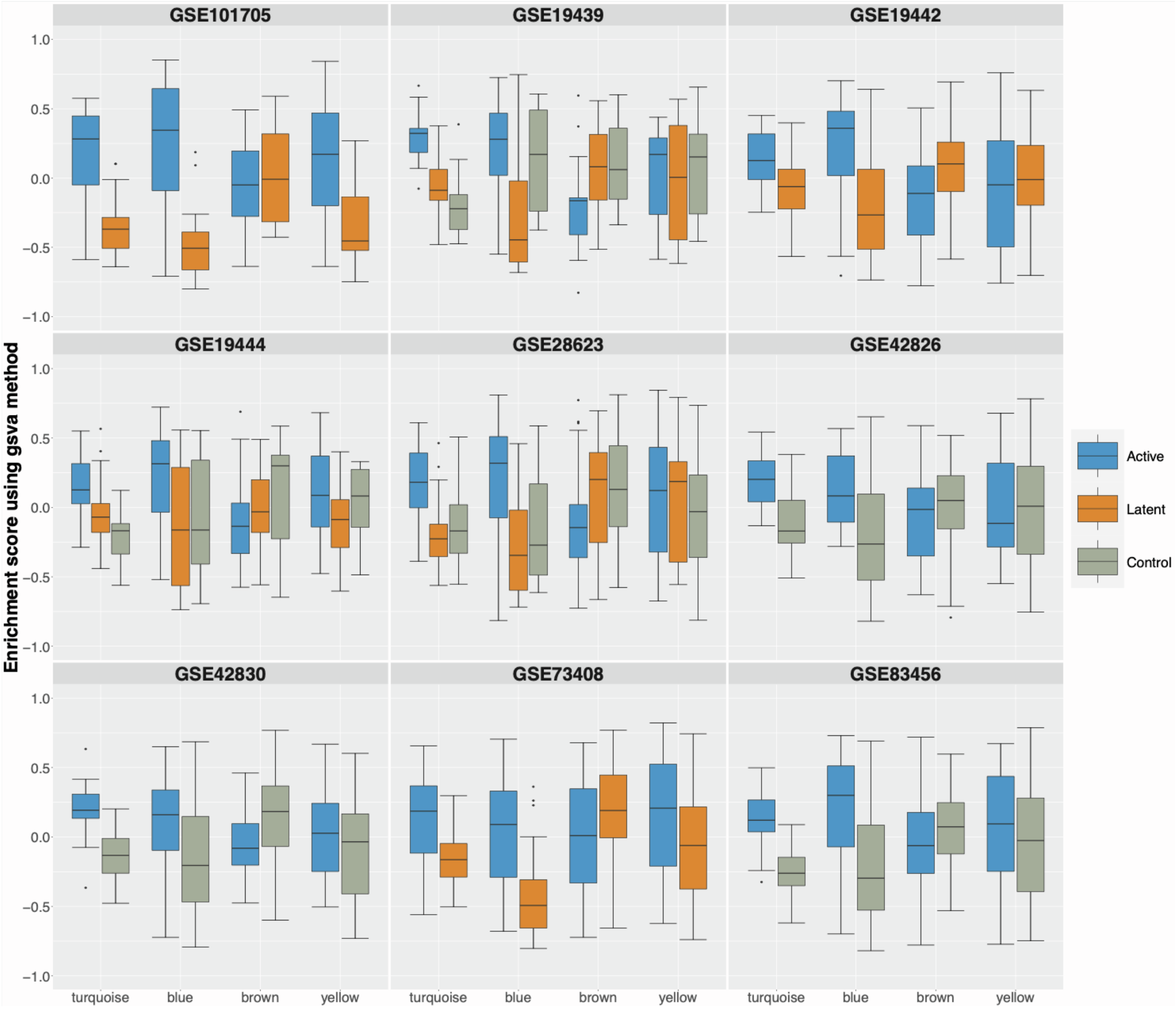

Figure S8. The enrichment analysis of all modules (from left to right: turquoise, blue, brown and yellow) on different transcriptomic datasets using the gsva method, Related to STAR Methods.

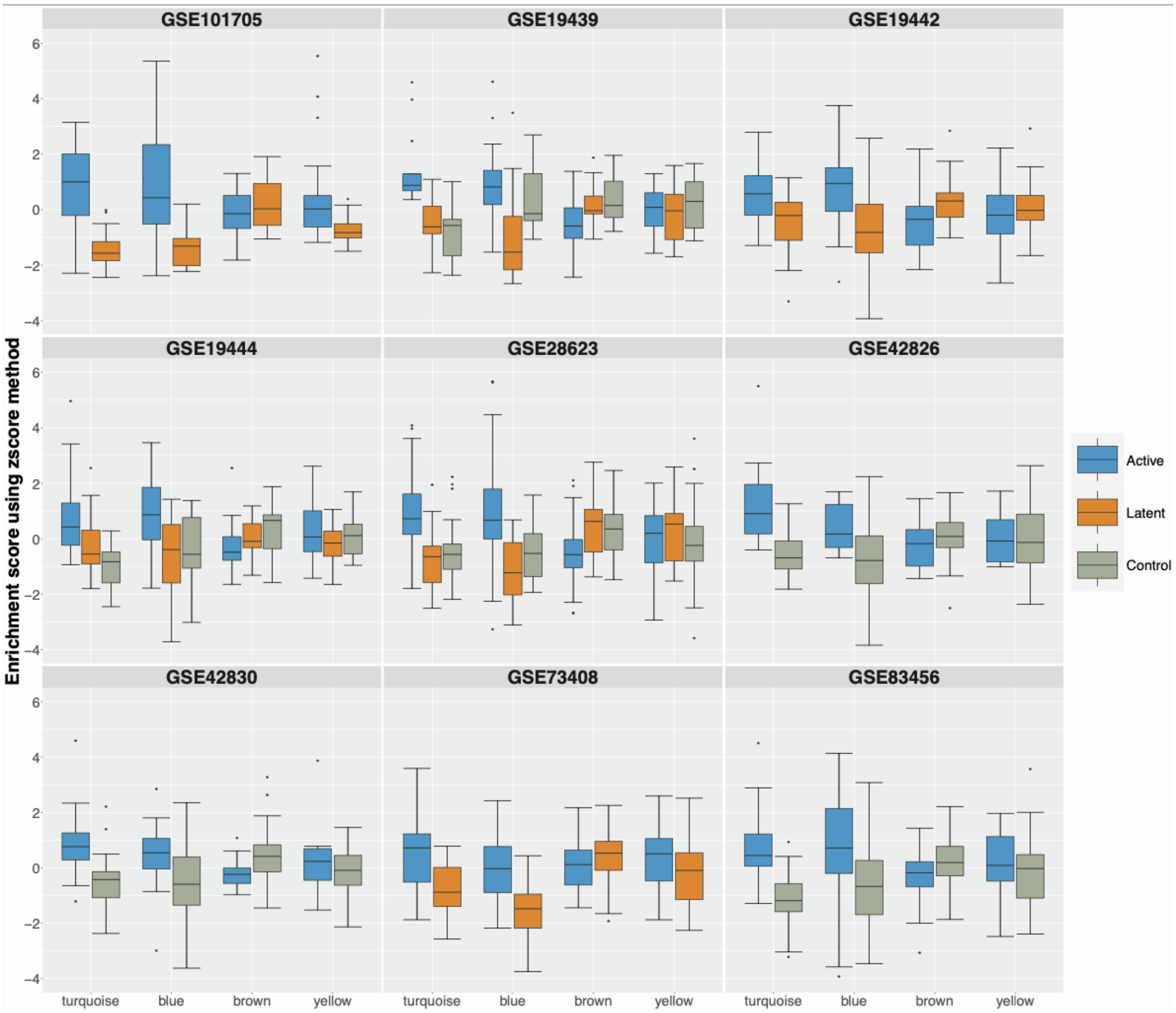

Figure S9. The enrichment analysis of all modules (from left to right: turquoise, blue, brown and yellow) on different transcriptomic datasets using the zscore method, Related to STAR Methods.

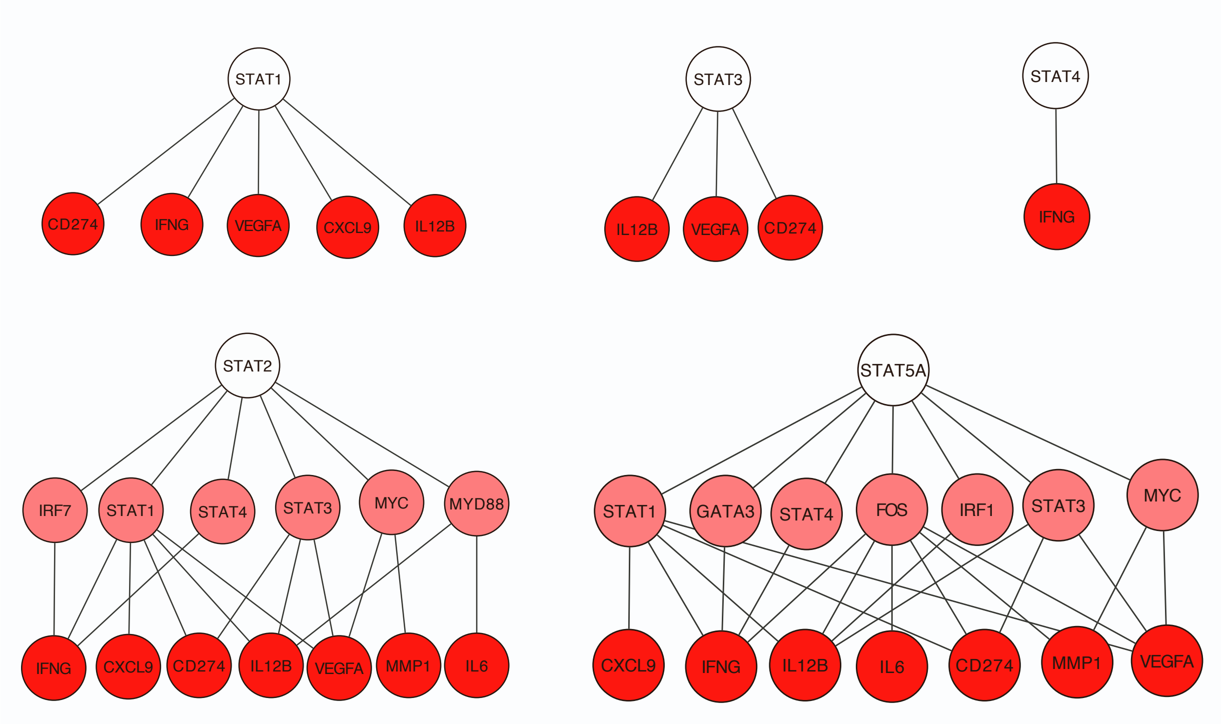

Figure S10. The signalling pathways between different signal transducer and activator of transcription (STAT) transcription factors and the proteins of our signature regarding the KEGG database, Related to Figure 4C.

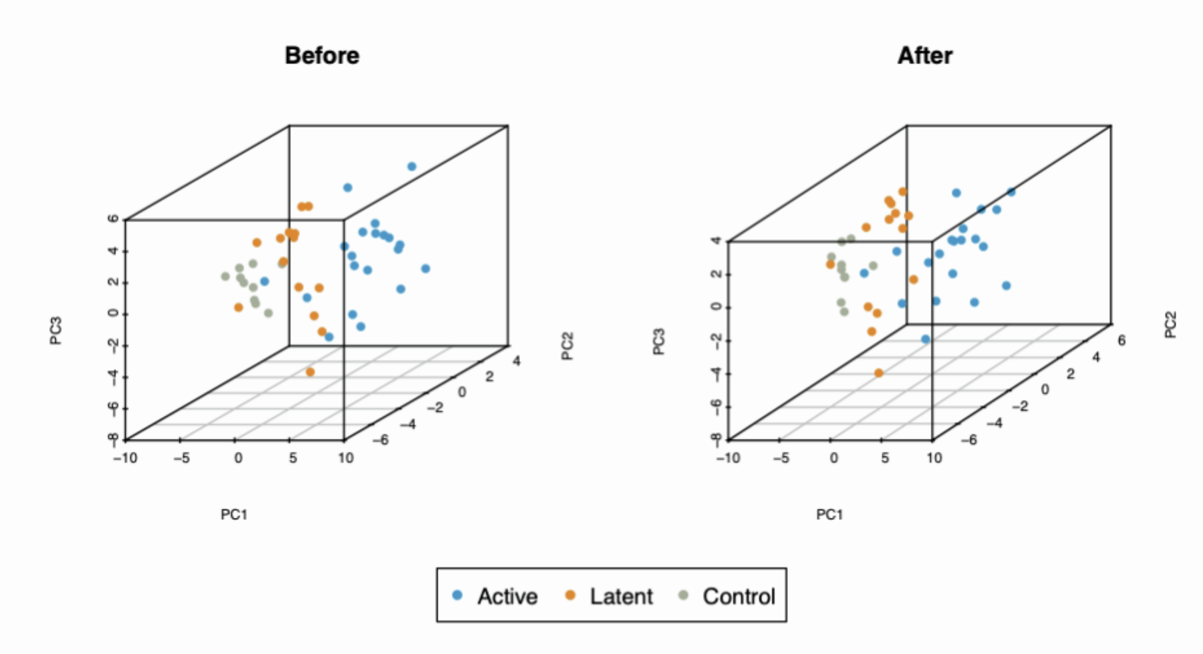

Figure S11. Visualizing samples of different groups: before and after batch effect removal, Related to STAR Methods.

Table S1. Details on selected transcriptomic datasets from the curatedTBData R package, related to Figure 4A.

Dataset	GeographicalRegion	Tissue	Age	HIVStatus	Control	Latent	Active
GSE73408	US	Whole Blood	>18	Negative	NA	35	35
GSE101705	South India	Whole Blood	>18	Negative	NA	16	28
GSE19439	UK	Whole Blood	19-72	Negative	12	17	13
GSE19442	South Africa	Whole Blood	18-48	Negative	NA	31	20
GSE19444	UK	Whole Blood	>18	Negative	12	21	21
GSE42826	Germany	Whole Blood	>17	Negative	52	NA	11
GSE42830	Germany	Whole Blood	>17	Negative	38	NA	16
GSE83456	UK	Whole Blood	NA	Negative	61	NA	45
GSE28623	The Gambia	Whole Blood	16-53	Negative	37	25	46

Table S2. Details of Active TB patients, Related to Figure 5.

**Active TB**	n=20	
**Symptoms**		
cough, n	12	
fever/night sweats, n	13	
duration, mo (mean, range)	4,5 (1-24)	
**Manifestation**		
pulmonary/pleuritis (+/-lgll)	16	
lymphnode	1	
disseminated	2	
Other*	1	
**CXR/CT**		
any pathology	17	
infiltrates	16	
cavities	15	
pleural effusion	3	
**Mycobacteriology**		
sputum microscopy pos	6	
any sample microscopy pos	6	
PCR positive	12	
culture positive	19	
**Biochemistry**	Mean (range)	n outside the reference interval
CRP	25 (1-94)	14 (>5)
ESR	54 (7-119)	15 (>20)
leukocytes	6.4 (3.2-12)	2 (>9.5)
hemoglobin	125 (102-149)	9 (F<120, M<130)
albumin	32 (26-38)	16 (<38)

*other= soft tissue abscess

Table S3. Plasma proteins analyzed by inflammation panel of Olink, related to STAR Methods.

Protein Name (Short Name)	Uniprot ID	Protein Name (Short Name)	Uniprot ID
Adenosine Deaminase (ADA)	P00813	Interleukin-20 receptor subunit alpha (IL-20RA)	Q9UHF4
Artemin (ARTN)	Q5T4W7	Interleukin-22 receptor subunit alpha-1 (IL-22 RA1)	Q8N6P7
Axin-1 (AXIN1)	O15169	Interleukin-24 (IL-24)	Q13007
Beta-nerve growth factor (Beta-NGF)	P01138	Interleukin-33 (IL-33)	O95760
Brain-derived neurotrophic factor (BDNF)	P23560	Latency-associated peptide transforming growth factor beta-1 (LAP TGF-beta-1)	P01137
Caspase-8 (CASP-8)	Q14790	Leukemia inhibitory factor (LIF)	P15018
C-C motif chemokine 3 (CCL3)	P10147	Leukemia inhibitory factor receptor (LIF-R)	P42702
C-C motif chemokine 4 (CCL4)	P13236	Macrophage colony-stimulating factor 1 (CSF-1)	P09603
C-C motif chemokine 19 (CCL19)	Q99731	Matrix metalloproteinase-1 (MMP-1)	P03956
C-C motif chemokine 20 (CCL20)	P78556	Matrix metalloproteinase-10 (MMP-10)	P09238
C-C motif chemokine 23 (CCL23)	P55773	Monocyte chemotactic protein 1 (MCP-1)	P13500
C-C motif chemokine 25 (CCL25)	O15444	Monocyte chemotactic protein 2 (MCP-2)	P80075
C-C motif chemokine 28 (CCL28)	Q9NRJ3	Monocyte chemotactic protein 3 (MCP-3)	P80098
CD40L receptor (CD40)	P25942	Monocyte chemotactic protein 4 (MCP-4)	Q99616
CUB domain-containing protein 1 (CDCP1)	Q9H5V8	Natural killer cell receptor 2B4 (CD244)	Q9BZW8
C-X-C motif chemokine 1 (CXCL1)	P09341	Neurotrophin-3 (NT-3)	P20783
C-X-C motif chemokine 5 (CXCL5)	P42830	Neurturin (NRTN)	Q99748
C-X-C motif chemokine 6 (CXCL6)	P80162	Oncostatin-M (OSM)	P13725
C-X-C motif chemokine 9 (CXCL9)	Q07325	Osteoprotegerin (OPG)	O00300
C-X-C motif chemokine 10 (CXCL10)	P02778	Programmed cell death 1 ligand 1 (PD-L1)	Q9NZQ7
C-X-C motif chemokine 11 (CXCL11)	O14625	Protein S100-A12 (EN-RAGE)	P80511
Cystatin D (CST5)	P28325	Signaling lymphocytic activation molecule (SLAMF1)	Q13291
Delta and Notch-like epidermal growth factor-related receptor (DNER)	Q8NFT8	SIR2-like protein 2 (SIRT2)	Q8IXJ6
Eotaxin (CCL11)	P51671	STAM-binding protein (STAMBP)	O95630
Eukaryotic translation initiation factor 4E-binding protein 1 (4E-BP1)	Q13541	Stem cell factor (SCF)	P21583
Fibroblast growth factor 21 (FGF-21)	Q9NSA1	Sulfotransferase 1A1 (ST1A1)	P50225
Fibroblast growth factor 23 (FGF-23)	Q9GZV9	T cell surface glycoprotein CD6 isoform (CD6)	Q8WWJ7
Fibroblast growth factor 5 (FGF-5)	Q8NF90	T-cell surface glycoprotein CD5 (CD5)	P06127
Fibroblast growth factor 19 (FGF-19)	O95750	Thymic stromal lymphopoietin (TSLP)	Q969D9
Fms-related tyrosine kinase 3 ligand (Flt3L)	P49771	TNF-beta (TNFB)	P01374
Fractalkine (CX3CL1)	P78423	TNF-related activation-induced cytokine (TRANCE)	O14788
Glial cell line-derived neurotrophic factor (GDNF)	P39905	TNF-related apoptosis-inducing ligand (TRAIL)	P50591
Hepatocyte growth factor (HGF)	P14210	Transforming growth factor alpha (TGF-alpha)	P01135
Interferon gamma (IFN-gamma)	P01579	Tumor necrosis factor (Ligand) superfamily, member 12 (TWEAK)	O43508
Interleukin-1 alpha (IL-1 alpha)	P01583	Tumor necrosis factor (TNF)	P01375
Interleukin-2 (IL-2)	P60568	Tumor necrosis factor ligand superfamily member 14 (TNFSF14)	O43557
Interleukin-2 receptor subunit beta (IL-2RB)	P14784	Tumor necrosis factor receptor superfamily member 9 (TNFRSF9)	Q07011
Interleukin-4 (IL-4)	P05112	Urokinase-type plasminogen activator (uPA)	P00749
Interleukin-5 (IL5)	P05113	Vascular endothelial growth factor A (VEGF-A)	P15692
Interleukin-6 (IL6)	P05231		
Interleukin-7 (IL-7)	P13232		
Interleukin-8 (IL-8)	P10145		
Interleukin-10 (IL10)	P22301		
Interleukin-10 receptor subunit alpha (IL-10RA)	Q13651		
Interleukin-10 receptor subunit beta (IL-10RB)	Q08334		
Interleukin-12 subunit beta (IL-12B)	P29460		
Interleukin-13 (IL-13)	P35225		
Interleukin-15 receptor subunit alpha (IL-15RA)	Q13261		
Interleukin-17A (IL-17A)	Q16552		
Interleukin-17C (IL-17C)	Q9P0M4		
Interleukin-18 (IL-18)	Q14116		
Interleukin-18 receptor 1 (IL-18R1)	Q13478		
Interleukin-20 (IL-20)	Q9NYY1		
